# Revival of Zeolite‐Templated Nanocarbon Materials: Recent Advances in Energy Storage and Conversion

**DOI:** 10.1002/advs.202001335

**Published:** 2020-09-02

**Authors:** Jun Miao, Zhongling Lang, Tianyu Xue, Yan Li, Yiwen Li, Jiaji Cheng, Han Zhang, Zikang Tang

**Affiliations:** ^1^ Key Laboratory of Bioinorganic and Synthetic Chemistry (MOE) Institute of Applied Physics and Material Engineering University of Macau Taipa Macau SAR P. R. China; ^2^ Instituto de Ciencia de Materiales Madrid CSIC Madrid 28049 Spain; ^3^ Polyoxometalate Science of Ministry of Education Northeast Normal University Changchun Jilin 130024 P. R. China; ^4^ Institute of Microscale Optoelectronics Key Laboratory of Optoelectronic Devices and Systems of Ministry of Education and Guangdong Province College of Physics and Optoelectronic Engineering Shenzhen Key Laboratory of Micro‐Nano Photonic Information Technology Guangdong Laboratory of Artificial Intelligence and Digital Economy (SZ) Shenzhen University Shenzhen 518060 P. R. China; ^5^ Biodesign Center for Biosensors and Bioelectronics Biodesign Institute Arizona State University Tempe AZ 85281 USA; ^6^ Center for High Pressure Science State Key Laboratory of Metastable Materials Science and Technology Yanshan University Qinhuangdao 066004 P. R. China; ^7^ School of Material Science and Engineering Hubei University Wuhan 430062 P. R. China; ^8^ Department of Chemistry Purdue University West Lafayette IN 47907 USA

**Keywords:** electrocatalysis, nanocarbons, rechargeable batteries, supercapacitors, zeolites

## Abstract

Nanocarbon materials represent one of the hottest topics in physics, chemistry, and materials science. Preparation of nanocarbon materials by zeolite templates has been developing for more than 20 years. In recent years, novel structures and properties of zeolite‐templated nanocarbons have been evolving and new applications are emerging in the realm of energy storage and conversion. Here, recent progress of zeolite‐templated nanocarbons in advanced synthetic techniques, emerging properties, and novel applications is summarized: i) thanks to the diversity of zeolites, the structures of the corresponding nanocarbons are multitudinous; ii) by various synthetic techniques, novel properties of zeolite‐templated nanocarbons can be achieved, such as hierarchical porosity, heteroatom doping, and nanoparticle loading capacity; iii) the applications of zeolite‐templated nanocarbons are also evolving from traditional gas/vapor adsorption to advanced energy storage techniques including Li‐ion batteries, Li–S batteries, fuel cells, metal–O_2_ batteries, etc. Finally, a perspective is provided to forecast the future development of zeolite‐templated nanocarbon materials.

## Graphical abstract

The zeolite‐templating method is an effective strategy to synthesize and prepare nanocarbon materials with a variety of structures and morphologies. These nanocarbons can serve as models to elucidate the mechanisms of electrocatalysis, charging/discharging process of supercapacitors and secondary batteries, etc. and demonstrate potential applications in energy storage and conversion.

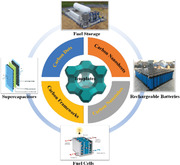

## 1 Introduction

In the 1980s, the discovery of fullerenes by Kroto and Smalley^[^ ^1^ ^]^ excited and pleased chemists; in the 1990s, the preparation of carbon nanotubes (CNTs) by Iijima^[^ ^2^ ^]^ delighted physicists; in the new millennium, pioneering work by Novoselov et al.^[^ ^3^ ^]^ on graphene charged up materials scientists; recently, the theoretical generation of carbon schwarzites via zeolite‐templating has aroused enthusiasm of scientists to explore novel allotropes of carbon.^[^ ^4^, ^5^ ^]^ For more than three decades, nanocarbon materials have provided scientific and technological excitement for scientists working in various disciplines.^[^ ^6^, ^7^, ^8^ ^]^

Carbon is one of the most fascinating chemical elements, since the carbon atom with four valence electrons can form robust bonds in different modes. By hybridization of sp, sp^2^, and sp^3^ hybridization, carbon elements can construct diverse molecular structures, which can form distinct‐different carbon allotropes such as diamonds,^[^ ^9^ ^]^ graphite,^[^ ^10^, ^11^ ^]^ and graphdiyne.^[^ ^12^, ^13^, ^14^ ^]^ Diverse hybridizations lead to totally different structures, morphologies, and properties: C‐C sp^3^ hybridization in diamond structure makes it an electrical insulator and the hardest material in nature;^[^ ^15^ ^]^ conversely, hybridization of sp^2^ makes graphite layered structure with loose interlamellar coupling, which can be mechanically exfoliated to prepare graphene with high conductivity; unique sp‐sp^2^ hybridizations could generate graphdiyne, a new 2D carbon allotrope.^[^ ^12^, ^13^, ^14^ ^]^ In the last three decades, a burgeoning number of novel carbon nanostructures including carbon dots (CDs),^[^ ^16^ ^]^ CNTs,^[^ ^17^ ^]^ graphene,^[^ ^18^ ^]^ graphdiyne,^[^ ^12^, ^13^, ^14^ ^]^ graphene‐like porous carbons,^[^ ^19^, ^20^, ^21^ ^]^ carbon nanocages,^[^ ^22^ ^]^ etc., were reported based on abovementioned sp, sp^2^, and sp^3^ hybridizations with their unique structures and novel properties. Therefore, the nanocarbon materials have attracted surge of interest from not only academia but also industry because of potential applications in catalysis,^[^ ^23^ ^]^ energy,^[^ ^24^ ^]^ environment,^[^ ^25^ ^]^ sensors,^[^ ^26^, ^27^, ^28^ ^]^ optoelectronic devices,^[^ ^29^, ^30^, ^31^, ^32^ ^]^ etc.

Crystalline porous materials with defined molecular‐level structures such as zeolites,^[^ ^33^, ^34^, ^35^, ^36^ ^]^ metal organic frameworks (MOFs),^[^ ^37^, ^38^, ^39^, ^40^ ^]^ and covalent organic frameworks (COFs)^[^ ^41^, ^42^, ^43^, ^44^ ^]^ are attracting increasing attention, which exhibit favorable properties such as molecule size recognition and selective catalysis. Additionally, the ordered array of porous structure can facilitate mass transfer.^[^ ^45^, ^46^, ^47^ ^]^ Furthermore, the frameworks can be readily and softly modified by means of chemical methods.^[^ ^48^, ^49^, ^50^ ^]^ The hard template method is a powerful tool for producing carbon materials with precisely controlled structures at the nanometer level, which would be comparable to other chemical and physical methods. Compared with MOFs and COFs, zeolites without organic moiety exhibit unique advantages in high temperature and pressure resistance.^[^ ^51^, ^52^, ^53^ ^]^ Therefore, they can serve as hard templates in synthesis and preparation of nanocarbon materials by the methods of chemical vapor deposition, in situ pyrolysis of organic templates, and hydro/solvothermal methods.^[^ ^16^, ^54^, ^55^, ^56^ ^]^ By removal of the zeolite, it is possible to obtain nanocarbon materials with 0D, 1D, 2D, or 3D structures from their unique nanospace in templates.^[^ ^57^, ^58^, ^59^ ^]^ Until April of 2019, 248 different types of zeolites have been recorded by International Zeolite Association (IZA), which possess 0D pores, 1D channels, 2D space, and 3D connected pores. Pioneering attempts to synthesize nanocarbon materials in zeolites can be traced to the end of last century by several research groups including us.^[^ ^6^, ^60^, ^61^, ^62^, ^63^, ^64^, ^65^ ^]^ In 2000, our group discovered the world's smallest SWCNT fabricated in the AFI zeolite by accident.^[^ ^64^ ^]^ Subsequently, we investigated its one‐dimensional superconducting and anisotropic optical absorption properties in 2001.^[^ ^6^ ^]^ Nowadays, the family of zeolite templated nanocarbon materials has been extended to carbon dots, graphene nanoribbons, and other nanocarbon materials with a diversity of morphology and functionality. Their applications are not limited to superconductivity, adsorption, and so forth. In this decade, they have been also applied in energy storage and conversion with development of the diverse functionality of these nanocarbon materials, including improvement of synthesis methods, modification of morphology, heteroatom doping, etc.

The energy crisis and environment contamination are two great issues currently threatening humanity. It is becoming urgent topic for scientists to explore environmentally benign energy storage devices^[^ ^66^, ^67^, ^68^, ^69^, ^70^ ^]^ and look for renewable alternatives of traditional fuels.^[^ ^71^, ^72^ ^]^ Zeolite‐templated nanocarbons (ZTNCs), possessing remarkable mechanical, optical, electrical, and catalytic properties, endow them enormous potential as energy storage materials and electrocatalysts: more specifically, high specific surface area is beneficial for gas adsorption^[^ ^73^ ^]^ and improvement of double layer capacitance;^[^ ^55^, ^74^, ^75^, ^76^, ^77^, ^78^, ^79^ ^]^ the zeolite‐templated carbon frameworks with high conductivity are also suitable candidates as electrode materials^[^ ^55^, ^73^, ^74^, ^77^, ^79^, ^80^, ^81^, ^82^, ^83^, ^84^, ^85^, ^86^, ^87^, ^88^, ^89^, ^90^ ^]^ and promising scaffolds for electrocatalysts;^[^ ^81^, ^91^, ^92^, ^93^, ^94^, ^95^, ^96^ ^]^ hierarchically ordered porosity can enhance mass transfer without pore blockage,^[^ ^78^, ^91^, ^97^, ^98^, ^99^ ^]^ which has already been employed as the host of active materials in electrode;^[^ ^83^, ^85^, ^100^ ^]^ plus ultrafine nanostructures anchored on zeolite‐templated nanocarbons, enhanced electrocatalytic performance can be implemented by synergic effect of nanocarbons and metal nanostructures.^[^ ^81^, ^91^ ^]^

**Figure 1:**
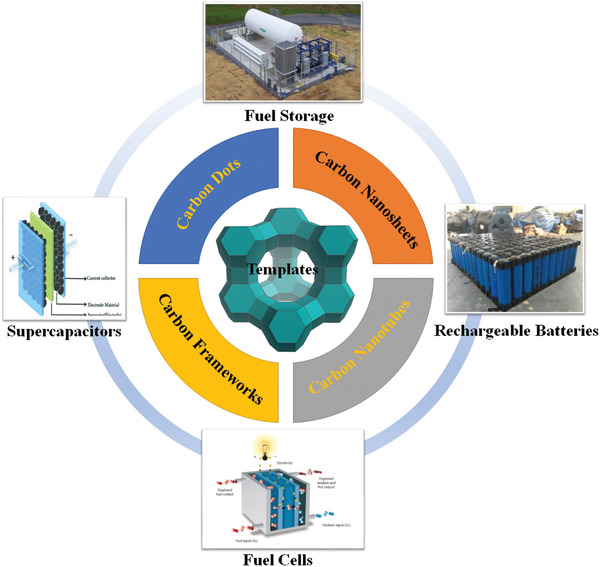
Schematic illustration of nanocarbon materials templated by zeolites including: i) carbon dots, ii) carbon nanotubes, iii) carbon nanosheets, iv) carbon frameworks; and state‐of‐art applications of nanocarbon materials in energy storage and conversion, including: i) fuel storage (hydrogen adsorption, methane adsorption), ii) supercapacitors, iii) rechargeable batteries (metal ion batteries, metal–O_2_ batteries, Li–S batteries, etc.), and iv) fuel cells.

The definition of nanocarbons was proposed by Inagaki et al.,^[^ ^101^, ^102^ ^]^ which contemplates not only the control of size but also the control of structure and texture at the nanoscale. In this review, ZTNCs refer to nanosized carbons (CDs, CNTs, and nanoribbons; graphene‐based nanosheets) and nanostructured porous carbons templated by zeolites. Herein, recent advances in zeolite‐templated nanocarbon materials are summarized from the aspect of synthesis, structures and their applications in energy storage and conversion. Actually, there already exist some reviews, which are only limited to zeolite‐templated CDs or 3D porous carbons in early years.^[^ ^54^, ^55^, ^56^ ^]^ However, the developments for those relevant nanocarbon materials, such as CNTs, graphene‐based nanoribbons, graphene‐based nanosheets are not included in these reviews. In this article, a comprehensive review is also present on their neoteric applications related to energy storage and conversion such as fuel storage (hydrogen adsorption, methane adsorption), supercapacitors, rechargeable batteries (metal ion batteries, metal‐O_2_ batteries, Li‐S batteries, etc.), and fuel cells emerged in recent years (as demonstrated in **Figure** **1**).

## 2 Preparation of Nanocarbon Materials in Zeolites Templates

### 2.1 Brief Introduction of Zeolites Templates

Zeolite molecular sieves, a family of aluminosilicates and their analogues, are crystalline, highly porous materials, which were originally observed in 1756 and 248 framework types have been recorded by IZA to date.^[^ ^103^, ^104^, ^105^ ^]^ Herein, a brief introduction is provided from the following aspects of: i) structures, ii) Lewis acidity, and iii) ion‐exchange capacity for ease of understanding the merits of zeolites as templates for synthesis of nanocarbons.

The basic building unit of zeolite structure is the individual tetrahedra with center of Al^3+^, Si^4+^, or other small ions with electrovalence of IV or more.^[^ ^106^, ^107^, ^108^ ^]^ The secondary building unit (SBU) consists of selected geometric groupings of primary tetrahedra linked by oxygen bridge.^[^ ^109^, ^110^ ^]^ There are nine such building units, which can be used to describe all of the known zeolite structures. These secondary building units consist of 4, 6, and 8‐membered single rings, 4‐4, 6‐6, and 8‐8‐member double rings, and 4‐1, 5‐1, and 4‐4‐1 branched rings. These crystal structures are precisely defined by its unique porous structure in terms of its pore diameters, shapes and connectivity.^[^ ^111^ ^]^ The structures of zeolites are categorized according to the following two aspects:

1. One provides internal pore system comprising: 1) small‐pore zeolites (MFI, LTA, etc.),^[^ ^112^, ^113^ ^]^ 2) medium‐pore zeolites (ZSM, etc.),^[^ ^114^, ^115^ ^]^ 3) large‐pore zeolites (FAU, EMT, etc.);^[^ ^116^, ^117^, ^118^ ^]^
2. The second specie provides a system of uniform pore dimensions^[^ ^119^, ^120^ ^]^ which, for instances, are: 1) 0D channel of zeolites (MTN, etc.),^[^ ^121^ ^]^ 2) 1D channel of zeolites (AEL, AFI, LTL, etc.),^[^ ^122^, ^123^ ^]^ 3) 2D channel of zeolites (HEU, IWT, MWW, etc.),^[^ ^124^, ^125^ ^]^ and 4) 3D channel of zeolites (CHA, BEA, etc.).^[^ ^126^, ^127^ ^]^

The zeolite pores can be adjusted to precisely determined uniform openings, hence, the molecules smaller than its pore diameter are allowed to be adsorbed, including organics such as ethylene and acetylene, namely “molecule sieving.” Significantly, the pores in many zeolites have diameters and shapes appropriate to accommodating fullerene, carbon nanotubes, and other nanocarbon materials.

**Figure 2:**
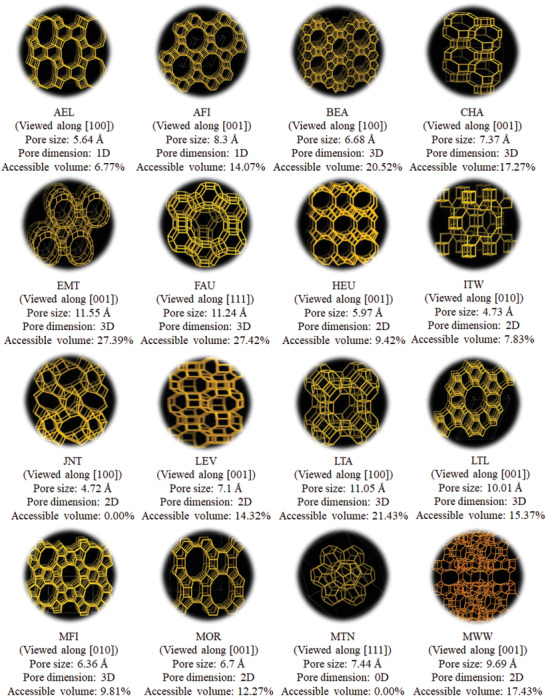
Sixteen types of zeolites employed as templates to prepare nanocarbon materials. Reproduced with permission; Copyright 2017, Structure Commission of the International Zeolite Association (IZA‐SC) (C. Baerlocher, L. B. McCusker, Database of Zeolite Structures: http://www.iza-structure.org/databases/).

The structure of zeolites has significant effect on synthesis and preparation of nanocarbon materials. This is because the size of pores and openings in zeolites determines the selections of carbon source. Additionally, the shape and connectivity of pores in zeolites, to a certain extent, meet the requirement of formation of CDs, CNTs, graphene, and 3D graphene‐like carbons. To date, there have been 16 types of zeolites employed as templates to prepare nanocarbon materials, as demonstrated in **Figure** **2**.

#### 2.1.1 Lewis Acidity

Lewis acidity of zeolites has been widely used in heterogeneous acid catalysis.^[^ ^128^, ^129^, ^130^, ^131^ ^]^ The Lewis acidity originates from substituted Al (III) in the frameworks of zeolites. In the process of zeolite‐templated methods, polycyclic aromatic compounds are liable to deposit on the acidic sites,^[^ ^132^ ^]^ which has positive effect on the formation of nanocarbon structures inside the nanochannels of zeolites. Hence, relatively high Al content, namely low Si/Al ratio is essential for the synthesis of nanocarbon materials templated by zeolites. Also, the uniformity of Lewis acid sites is beneficial for avoiding generation of coke, which gradually covers the acidic sites, degrades their catalytic activity and block the entrance to inner space of zeolites.^[^ ^133^, ^134^ ^]^

#### 2.1.2 Ion‐Exchange Capacity

Another important property of zeolites is ion‐exchange.^[^ ^135^, ^136^ ^]^ In zeolite framework, the negative charge on alumina tetrahedra is compensated by exchangeable cations resulting in an electrically neutral framework. The exchangeable cations, generally from the group I or II ions, can be exchanged with other cations through a solution‐based conventional ion‐exchange process.

In the process of synthesizing nanocarbon materials templated by zeolites, high temperature is indispensable to fix the carbon source inside the pores. Moreover, such high temperature simultaneously lower selectivity of the reactions. This problem can be tackled by ion‐exchange capacity of zeolites. In principle, transition metal ion can bond with carbon source, such as olefins, alkynes, and aromatic compounds by d–*π* coordination, which stabilize carbon source and formed reactive intermediates in the pores of zeolites.^[^ ^137^ ^]^ Thus, the catalytic active sites introduced by ion‐exchange can not only reduce the reaction conditions but enhance the selectivity of the products.

Above all, zeolite‐templated method exhibits promising catalytic properties to synthesize and prepare nanocarbon materials with extensive shape selectivity, which arise from synergistic effect among their Brønsted and Lewis acidity, ion‐exchange capacity, and molecule sieving. Specifically, synthesis of nanocarbon materials by the methods of chemical vapor deposition (CVD) and pyrolysis requires harsh reactive conditions and has the disadvantages such as low selectivity. Served as templates, zeolites have the properties of ion‐exchange capacity, molecular sieving but above all their wide‐ranging shape selective catalytic properties, which is helpful in moderating reactive conditions and improving selectivity. From the above, zeolites have attracted tremendous attention as template to synthesize nanocarbon materials in recent years.

### 2.2 Synthetic Methods

**Figure 3:**
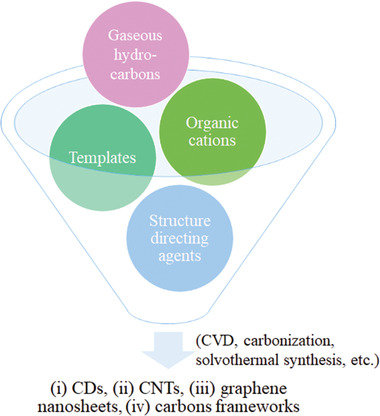
Carbon source, synthetic methods, and products of zeolite‐templated nanocarbons: i) CDs, ii) CNTs, iii) graphene nanosheets and iv) carbon frameworks.

Synthetic methods for zeolite templated nanocarbons (including CDs, CNTs, graphene, porous carbons, etc.) with tunable size, morphology, and functionalization can be generally classified into three main groups: i) CVD,^[^ ^84^, ^138^, ^139^ ^]^ ii) carbonization,^[^ ^61^, ^140^ ^]^ in situ pyrolysis of organic templates or loaded organic molecules, and iii) solvothermal/hydrothermal synthesis.^[^ ^141^ ^]^ As for CVD, gaseous hydrocarbons are employed as carbon source, such as ethylene and acetylene. Regarding carbonization, organic cations, templates, and structure directing agents of zeolites are desirable as carbon source for achieving successful carbonization within the zeolite pores. Besides, ionic liquid is also a promising candidate as carbon source, which can be classified as organic cations. Carbon source, synthetic methods and products described above are demonstrated in **Figure** **3**. Before pyrolysis, carbon impregnation is the key step, which can be divided in vapor phase method and liquid phase method. Specifically, the approaches of carbon impregnation include: i) vacuum impregnation technique,^[^ ^60^ ^]^ ii) soaking method or ion exchange,^[^ ^142^ ^]^ and iii) high‐pressure impregnation technique.^[^ ^74^ ^]^ In this section, post‐synthesis is also summarized including: i) chemical methods^[^ ^143^, ^144^, ^145^ ^]^ such as oxidation, fluorination and ii) electrochemical methods.^[^ ^146^, ^147^, ^148^, ^149^ ^]^ Herein, synthetic methods of zeolite‐templated nanocarbons are discussed from the aspects of: i) CVD, ii) in situ pyrolysis, iii) solvothermal synthesis, and iv) post‐synthesis.

#### 2.2.1 CVD Methods

CVD is a process in which the substrate is exposed to one or more volatile precursors, which react and/or decompose on the substrate surface to produce the desired deposits. CVD method is widely used for production of nanocarbon materials because it can decrease the temperature of reactions and increase large‐scale production of nanocarbon materials and allows control over the alignment, density, and diameter of nanocarbon materials.^[^ ^150^ ^]^ In this case, zeolites can serve as substrate owing to their high specific surface area, well‐organized and diverse nanochannel and catalytic properties. In addition, small molecules, such as ethylene and acetylene, can serve as volatile precursors. Zeolite as catalyst can decrease the temperature of reactions and increase selectivity of reactions. Moreover, doped nanocarbon materials can be obtained by using heteroatom‐containing volatile precursors, such as acetonitrile.^[^ ^138^ ^]^ Meanwhile, ionic liquid, such as 1‐ethyl‐3‐methylimidazolium tetracyanoborate (EMIT), is also employed as a boron and nitrogen source by carbonization method.^[^ ^142^ ^]^ Furthermore, structural diversity of zeolite allows structural and morphological control of nanocarbon materials. The process of uptaking carbon into nanochannels of zeolites by the method of CVD can be proposed by Nishihara et al. in the following three steps: Step I: rapid carbon diffusion from zeolite particles surface to their inner space; Step II: gradual increase of carbon density at a constant rate; and Step III: deposition of carbon is terminated.^[^ ^151^ ^]^ Herein, two examples are exhibited to elucidate how the parameters of CVD, such as: i) reaction temperature, ii) reaction duration, influence the formation of nanocarbon materials.

**Figure 4:**
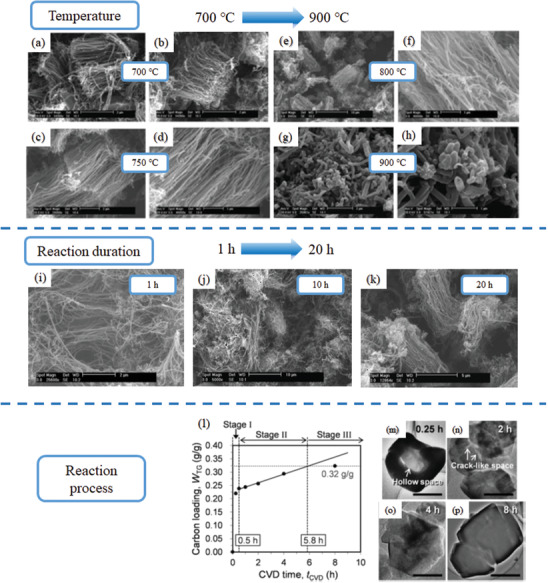
a–h) Influence of temperature and reaction duration on synthesis of zeolite‐templated nanocarbons: SEM images of CNTs, using NH_4_‐zeolite *β* as template and acetonitrile as carbon source, prepared at various CVD temperatures: a,b) 700 °C; c,d) 750 °C; e,f) 800 °C; g,h) 900 °C. i–l) SEM images of carbon nanotubes prepared via CVD at 800 °C using NH_4_‐zeolite *β* as substrate and acetonitrile as carbon source for various CVD durations: i) 1 h; j) 10 h; k) 20 h; l) the relation between W_TG_ and t_CVD_. m–p) Corresponding TEM images of liberated carbons in the particles of zeolite at t_CVD_ of 0.25, 2, 4, and 8 h. Scale bars are 1 µm. a–l) Reproduced with permission.^[^ ^63^ ^]^ Copyright 2005, American Chemical Society. m–p) Reproduced with permission.^[^ ^151^ ^]^ Copyright 2017, J‐STAGE.

Temperature has huge effect on structure and morphology of zeolite‐templated nanocarbon materials via CVD method. High temperature is beneficial for mass transfer and improves possibility of doping heteroatoms, whereas detrimental for the selectivity of the reaction generating structural defects and morphological random orientation. In this field, Mokayaet al. have reported a great deal of zeolite‐templated nanocarbon materials by CVD method. An example of N‐doped CNTs from Mokaya et al. was selected to elucidate temperature and reaction duration effect on preparation of zeolite‐templated nanocarbons. The synthesis of N‐doped CNTs was presented by CVD method using NH_4_‐zeolite *β* as the substrate, ferric nitrate as the catalyst, and acetonitrile as the carbon precursor.^[^ ^63^ ^]^ The influence of temperature on the morphology of N‐doped CNTs was also investigated with detail. After treatment with HF and refluxing in HCl, aligned N‐doped CNTs are synthesized after 20 h CVD duration at 700, 750, and 800 °C. As shown in top section of **Figure** **4**, the sample, which was prepared at 900 °C, presents randomly oriented and broken nanotubes. The length of the aligned CNTs increases with the increasing temperature (over the temperature range 700–800 °C). The sample prepared at 900 °C possesses various lengths, which also has much larger diameter than that of the aligned CNTs prepared at lower CVD temperature. Furthermore, the amounts of nitrogen of the samples also depend on the CVD temperature, which vary between 6 and 9 wt%. With regards to reaction duration, a series of N‐doped CNTs were prepared via similar method at 800 °C except the length of CVD durations (1, 10, or 20 h). As‐prepared N‐doped CNTs tend to arrange with better alignment after reacting for longer time, because the walls of N‐doped CNTs grow more robust with longer reaction duration, as shown in Figure 4i–k.

The structure and morphology are closely related to CVD period, mainly originating from the carbon loading process inside and outside the zeolite templates. Recently, the influence from CVD duration on zeolite templated nanocarbon materials was systematically investigated by Nishihara et al. A series of nanographenes were prepared using zeolite X as template, mixture gas of acetylene, and N_2_ with volumetric ratio of 15% at 600 °C.^[^ ^151^ ^]^ The amount of carbon loaded onto the zeolite template (W_TG_ [g‐carbon/g‐zeolite]) was measured by thermogravimetric analysis. The relation between W_TG_ and CVD period (t_CVD_) is demonstrated in Figure 4l of bottom section. Combined with TEM images, carbon filling process during CVD was analyzed with detail. The carbon is rapid deposited into empty zeolite pores in the initial 0.5 h. The corresponding TEM image shows a hollow space in the particle of zeolite. From 0.5 to 5.8 h, the rate of deposition become slower than that of Stage I because of the narrow pores of zeolite. Some crack‐like space appeared in the particle of zeolite at 2nd h, as shown in Figure 4n. Subsequently, the crack‐like space became smaller and then vanished gradually, as shown in Figure 4o. After reacting for 8 h, the deposition of carbon in the particle of zeolite looks homogenous, indicating that carbon is uniformly distributed in the pores of zeolite, as shown in Figure 4p. Since acetylene is not decomposed in a gas phase at the CVD temperature of 600 °C, acetylene will be converted into carbon only when existence of zeolite as catalyst. Hence, the process of carbon deposition will be accomplished when active sites of zeolite are completely covered by carbon. Consequently, Nishihara et al. speculate the process of carbon deposition is over at 5.8 h by extrapolation method, as shown in Figure 4i.

#### 2.2.2 Carbonization: In Situ Pyrolysis of Organic Templates or Loaded Organic Molecules

**Figure 5:**
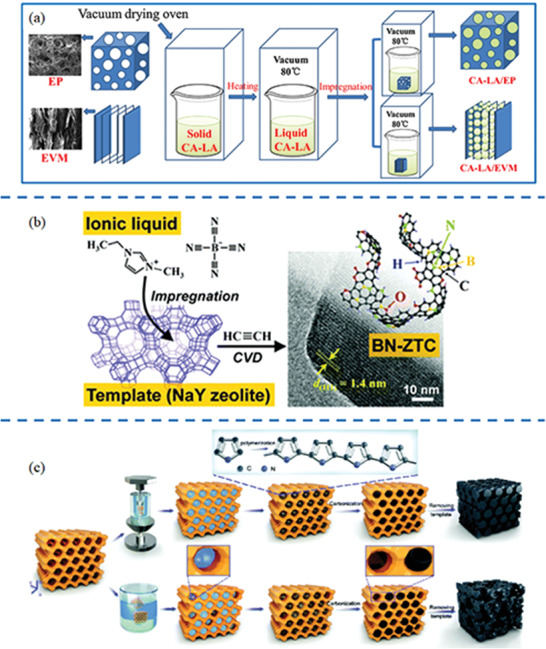
Schematic illustration of carbon introduction methods: a) Vacuum impregnation technique; b) soaking method or ion exchange; c) high‐pressure impregnation technique. a) Reproduced with permission.^[^ ^152^ ^]^ Copyright 2019, Elsevier B.V. b) Reproduced with permission.^[^ ^142^ ^]^ Copyright 2017, The Royal Society of Chemistry. c) Reproduced with permission.^[^ ^74^ ^]^ Copyright 2018, The Royal Society of Chemistry.

Zeolites and molecular sieves with well‐defined nano‐pores are ideal hosts to accommodate guests including organic templates, structure directing agents, loaded organic molecules, and organic cations. Thereby, the location and position of the guests inside the nano‐pores of the zeolite is strictly defined. Before pyrolysis, carbon impregnation is the key step, which can be divided in vapor phase and liquid phase methods. Specifically, the approaches of carbon impregnation include: i) vacuum impregnation technique,^[^ ^60^ ^]^ ii) soaking method or ion exchange,^[^ ^142^ ^]^ and iii) high‐pressure impregnation technique.^[^ ^74^ ^]^ As for liquid organics, they can be readily introduced into the pores of scaffolds by vacuum impregnation technique.^[^ ^60^, ^152^ ^]^ (**Figure** **5**a.) With regard to anodic framework of zeolite, cationic carbon source such as ionic liquids and small organic salts ammonium can be impregnated by soaking method or ion exchange, as shown in Figure 5b.^[^ ^142^, ^153^ ^]^ Recent development of high‐pressure impregnation technique^[^ ^74^, ^154^ ^]^ endowed zeolite‐templated nanocarbon higher specific surface area and enhanced structural ordering in comparison with conventional methods, as shown in Figure 5c.

Pyrolysis of guests in zeolites and molecular sieves is one of the most important methods for preparing well dispersed nanocarbon materials. Zeolites/molecular sieves have been employed as matrix to prepare nanocarbon materials by in situ pyrolysis of organic templates or loaded organic molecules, especially carbogenic nanodots (denoted as CNDs) with narrow size distribution in recent 10 years.^[^ ^155^, ^156^, ^157^, ^158^ ^]^ The pioneering work was first reported by Bourlinos et al.^[^ ^155^ ^]^ The synthetic method of supported carbon dots was described as thermal oxidation of the zeolite‐hosted organic species by appropriate ion‐exchange, which results in near spherical carbogenic nanoparticles grafted onto the external surfaces of the NaY zeolite. In this case, the cation, protonated 2,4‐diaminophenol, is too large to access into the internal space of zeolite, which limits synthesis of well dispersed nanocarbon materials. Continuously, Chen et al. developed a series of carbogenic nanoparticles, which were confined in MAPO‐44 (CHA), by pyrolysis of either template reagents or loaded organic molecules.^[^ ^157^ ^]^ Thus, as‐prepared products possessed well‐dispersion in comparison with previous reports.

Baldovi et al. employed small pore zeolites, ITQ‐29 (LTA structure), and ITQ‐12 (ITW structure) as rigid templates, tetramethylammonium hydroxide (TMAOH), 4‐methyl‐2,3,6,7‐tetrahydro‐1H,5H‐pyrido[3.2.1‐ij]quinolinium hydroxide, and 1,3,4‐trimethylimidazolium as the organic structure directing agent respectively.^[^ ^159^ ^]^ After compressing to high pressure of 3 Ton cm^−2^, these pelletized zeolite samples were calcined in inert atmosphere at required temperature. Carbon dots with particle size from 5 to 12 nm were obtained after etching the matrix of ITQ‐29 and ITQ‐12. However, it is unsuccessful to prepare carbon dots when extending this methodology to medium or large pore zeolites, such as silicalite (MFI structure) or zeolite *β* (BEA structure). The failure was imputed to the synergetic effect of both geometry of the pores in zeolites and the nature of the quaternary ammonium cation used as the templates.

#### 2.2.3 Solvothermal/Hydrothermal Synthesis

**Figure 6:**
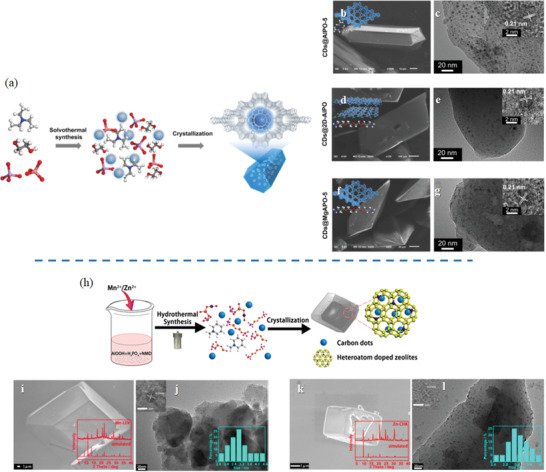
Solvothermal/hydrothermal synthesis of zeolite‐templated nanocarbons: a) Schematic illustration of formation of CDs in AlPO‐5; b,d,f) SEM images of CDs@ AlPO‐5, CDs@2D‐ AlPO, and CDs@MgAPO‐5 (inset: structures of SDAs and zeolites); c,e,g) TEM images of CDs@ AlPO‐5, CDs@2D‐ AlPO, and CDs@MgAPO‐5 (inset: HRTEM images). h) Schematic illustration of formation of CDs in heteroatom doped zeolites; i,k) SEM images of CDs@Mn‐LEV and CDs@Zn‐CHA (inset: XRD spectrums); j,l) TEM images of CDs@Mn‐LEV and CDs@Zn‐CHA (inset: size distributions of CDs). a–g) Reproduced with permission.^[^ ^141^ ^]^ Copyright 2017, The Authors, published by AAAS. Reprinted/adapted from ref. [141]. © The Authors, some rights reserved; exclusive licensee American Association for the Advancement of Science. Distributed under a Creative Commons Attribution NonCommercial License 4.0 (CC BY‐NC) http://creativecommons.org/licenses/by‐nc/4.0/. h–l) Reproduced with permission.^[^ ^160^ ^]^ Copyright 2019, American Chemical Society.

In general, solvothermal/hydrothermal synthesis is adopted to prepare nanocarbon materials and matrix simultaneously in one system.^[^ ^141^, ^160^ ^]^ Li et al. first proposed the concept of in situ formation process of carbon dots (CDs) in zeolites.^[^ ^141^, ^160^ ^]^ By using solvothermal method, a series of CDs were prepared accompanied with the crystallization of zeolite in one‐pot reaction, in which the CDs can be in situ encapsulated and confined in the zeolite matrix (**Figure** **6**a,h). By using various organic structure‐directing agents (SDAs), CDs embedded in various zeolite matrix were obtained. Triethylamine (TEA) and 4,7,10‐trioxa‐1,13‐tridecanediamine (TTDDA) were employed as SDAs respectively. In the former reaction, hexagonal prism‐like crystals of AlPO‐5 was prepared simultaneously with encapsulated CDs, as shown in Figure 6b,c. In the latter reaction, as‐prepared product displayed plate‐like crystals of UT‐5 with CDs embedded in the layered structure, as demonstrated in Figure 6d,e. By using the same SDA, CDs@MgAPO‐5 was successfully prepared in the hydrothermal reaction system of Al(O*i*Pr)_3_‐MgHPO_4_·3H_2_O‐H_3_PO_4_‐TTDDA‐H_2_O. As shown in Figure 6f,g, the SEM and TEM images of as‐obtained crystals, which is isostructural with the AFI structure, displays polyhedral morphology with well‐dispersed CDs. CDs encapsulated by heteroatom doped zeolites, CDs@Mn‐LEV, and CDs@Zn‐CHA, were successfully prepared by similar method (Figure 6h). In this case, *N*‐methylpiperidine (NMD) served as both organic template and precursor. After hydrothermal reaction at 180 °C for 3 days, cuboid‐like and cubic crystals were obtained respectively. Compared with XRD spectrums, SEM and TEM images showed that CDs were embedded into the pores of Mn‐LEV and Zn‐CHA (Figure 6i–l).

It is worth noting that nanocarbon materials in situ synthesized by solvothermal/hydrothermal synthesis is not easy to achieve. The prerequisite for solvothermal/hydrothermal synthesis is that synthetic conditions of both nanocarbon materials and zeolites are similar. Meanwhile, a strong driving force is essential to co‐assemble the nanocarbon materials and zeolite matrix. Thus, to date, solvothermal/hydrothermal synthesis of nanocarbon materials templated by zeolites remain limited in synthesis of CDs.

#### 2.2.4 Post‐Modification

Post‐synthetic surface functionalization gives new properties to nanocarbon materials, for instance, improving selective adsorption, boosting charge storage, and increasing catalytic sites. A diversity of methods has been generally applied to modify the amount and nature of surface functional groups on porous and nano‐sized carbon materials.^[^ ^161^ ^]^ Among them, conventional chemical and electrochemical methods are the two main methods.

##### Conventional Chemical Methods

Conventional chemical method is widely used for post‐functionalization of zeolite templated nanocarbon materials.^[^ ^162^ ^]^ It is essentially a chemical reaction in the gas or liquid phase with a suitable oxidizing or reducing agents and further heat treatment in an inert atmosphere. In general, the influence of these conventional oxidative treatments on the textural and structural properties, to a great extent, depends on the nature of the nanocarbon material and oxidizing agents introduce varying amounts of oxygen groups depending on their oxidizing ability, oxidant concentration, temperature, and time of treatment, hence, showing a relatively low selectivity.

**Figure 7:**
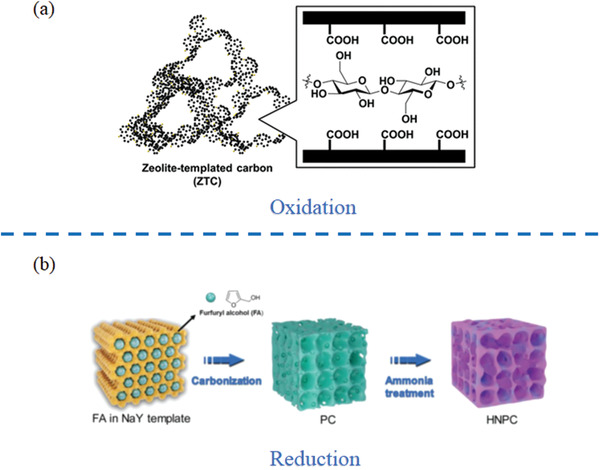
a) Schematic illustration of oxidic modification of zeolite‐templated nanocarbons; b) reductive modification of zeolite‐templated nanocarbons. a) Reproduced with permission.^[^ ^143^ ^]^ Copyright 2016, American Chemical Society. b) Reproduced with permission.^[^ ^154^ ^]^ Copyright 2019, John Wiley & Sons.

Fukuoka et al. developed a conventional chemical method for post‐modification of zeolite templated nanocarbon materials and systematically investigate their catalytical performance for hydrolysis of cellulose‐derived long‐Chain glucans^[^ ^143^ ^]^ (**Figure** **7**a). In this case, the weak acid sites, carboxylate functional groups, were introduced using either hydrogen peroxide, nitric acid, or sodium hypochlorite as an oxidant.^[^ ^144^, ^145^ ^]^ Among them, only the samples treated by hydrogen peroxide stay intact framework. Oppositely, the collapse of frameworks is common existing in the samples treated by nitric acid, or sodium hypochlorite. In the process of hydrogen peroxide treatment, the amount of carboxylate functional groups starts to increase dramatically and then tend to flatten out. As for post‐modification by reduction reaction, Lu et al. developed N‐doped porous carbon by ammonia treatment, as shown in Figure 7b.^[^ ^154^ ^]^ Ammonia treatment enabled both introduction of N‐containing functional groups and formation of hierarchical structures. Hence, it is crucial to adopt proper reductant/oxidant and treatment period for maintaining the framework intact and providing sufficient functionality.

##### Electrochemical Methods

In the process of conventional chemical methods, strict operation conditions are compulsory to prevent the framework of nanocarbon materials from collapse. In addition, low selectivity and uncontrollability of conventional chemical methods limit its applications. Interestingly, recent studies on the electrochemical modification of a granular activated carbon have revealed that the electrochemical method is a promising technique to attain a more selective and controlled modification of the surface chemistry of carbon materials.

**Figure 8:**
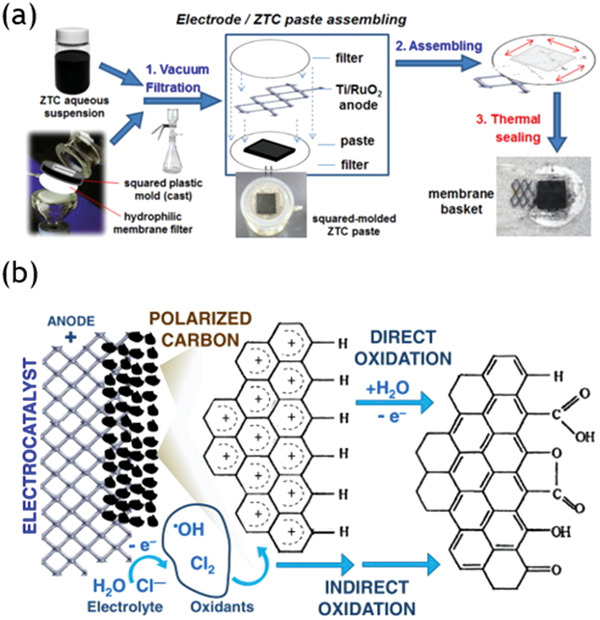
a) Electrode/sample paste assembling process for electrochemical oxidation; b) the process of electrochemical oxidation including direct oxidation and indirect oxidation. a,b) Reproduced with permission.^[^ ^148^ ^]^ Copyright 2012, Elsevier Ltd.

Previously, Morallón et al. investigated a series of electrochemical methods for post‐modification of nanocarbon materials.^[^ ^146^, ^147^ ^]^ One example of electrochemical modification of zeolite templated nanocarbon materials from them is anodically oxidation methods by galvanostatic polarization experiments.^[^ ^148^ ^]^ The home‐made electrode was adopted as working electrode prepared as demonstrated in **Figure** **8**a, commercial Pt electrode as a counter electrode, and Ag/AgCl electrode as a reference electrode. Surface functionality of the product such as the number of CO_2_‐evolving groups was determined by different conditions of current and time. The mechanism for the electrochemical oxidation of nanocarbon materials was proposed as the direct and indirect oxidation pathways, as shown in Figure 8b. The direct oxidation mechanism was that CO_2_‐evolving groups were generated on the surfaces of nanocarbon materials. In the indirect process, CO‐evolving groups were further oxidized by the hydroxyl radicals and Cl_2_, which was previously generated on the surfaces of Ti/RuO2 anode. Thus, the species of functional groups are determined by electrode potential while their amount depends on treatment period.

Another example of electrochemical methods for post‐modification of zeolite‐templated nanocarbon materials was reported by Cazorla‐Amorós et al.^[^ ^149^ ^]^ The preparation process was conducted through potentiodynamic treatment in acid media under oxidative conditions. Unique structures of zeolite‐templated nanocarbon materials were retained by limiting the potential of the potentiodynamic treatment in presence of perchloric acid and aminobenzene acids. The polymer chains were formed on the surface of zeolite‐templated nanocarbon materials along with covalently bonded functionalities. Nitrogen functional groups were successfully introduced by using two different aminobenzene acids: 2‐aminobenzoic (2‐ABA) and 4‐aminobenzoic acid (4‐ABA). To conclude, not only electrode potential determines the species of functional groups, selection of electrolytes also have a significant influence on functional groups.

Regularity of pores with plentiful edge sites is the crucial characteristics of zeolite‐templated nanocarbons. Thus, another issue that readership may concern is the characterization methods of edge sites. Relevant methods, including diffuse reflectance infrared Fourier transform (DRIFT) spectroscopy,^[^ ^143^ ^]^ X‐ray photoelectron spectroscopy (XPS),^[^ ^154^ ^]^ and temperature‐programmed desorption (TPD) method,^[^ ^146^ ^]^ have been reported to characterize the edge sites of zeolite‐templated nanocarbons. DRIFT spectroscopy is a technique that collects and analyzes scattered IR energy, which is used for measurement of fine particles and powders, as well as rough surface. In the case of nanocarbons, the oxygenated functional groups of zeolite‐templated nanocarbons were characterized by DRIFT spectroscopy.^[^ ^143^ ^]^ XPS is a method to determine the kinetic energy spectrum of photoelectrons ejected from the surface of a specimen by the irradiating X‐ray having a constant energy in vacuum, which is utilized for identification of an element and estimation of its chemical bonding state in the specimen. It has been widely employed to determine N‐containing functional groups in nanocarbons..^[^ ^23^, ^154^ ^]^ TPD technique is often used to monitor surface interactions between adsorbed molecules and substrate surface. Differed from traditional TPD spectroscopy, oxygen‐containing functional groups are thermally decomposed to release CO_2_, CO, and H_2_O at different temperatures, depending on the thermal stability of the groups in the case of nanocarbons. Thus, it is one of the powerful tools to characterize surface oxygen‐containing functional groups on carbon materials.

## 3 Structure and Morphology of Zeolite‐Templated Nanocarbons in Different Dimensions

As early as 20 years ago, Kyotani et al. first proposed the narrowly defined concept of zeolite‐templated carbons, a class of crystalline microporous carbons with ordered microporous frameworks and structure regularity, templated by zeolites.^[^ ^60^ ^]^ Twenty years later, Nishihara et al. proposed categories of zeolite‐templated carbons.^[^ ^54^ ^]^ They pointed out that there are three types of zeolite‐templated nanocarbons: type I is the nanocarbons with relatively ordered structures since carbon source merely deposits into the pores of zeolites and form replica of zeolite pores; type II is the nanocarbons with both ordered and disordered moieties since unfavored aggregation occurs without confinement of pores of zeolites; type III is the amorphous carbons since the carbon source only deposit on the outer surface of zeolite crystals. Meanwhile, Nishihara et al. added that Type‐III is intrinsically not zeolite‐templated carbons by strict definition.

Actually, carbon materials prepared by using zeolites as templates are not confined crystalline microporous carbons. Zeolites have a variety of pore structures including 1D, 2D to 3D structures; in addition, the pores of the zeolites are also connected in various ways. Hence, the prepared nanocarbon materials can be of various dimensions and various morphology. Moreover, different carbon impregnation methods, synthesis temperature and reaction time all influence the structure and morphology of synthesized nanocarbon materials. Besides, it is generally known that the dimension of materials has great influences on their physical and chemical properties.^[^ ^163^, ^164^, ^165^ ^]^ Thus, we define the generalized definition of nanostructured carbons templated by zeolites as “zeolite‐templated nanocarbons” including CDs, CNTs, carbon nanoribbons, graphene nanosheets as well as zeolite‐templated carbons. Herein, recent advances on structures and morphology zeolite‐templated nanocarbons of are summarized in aspects of different dimensions.

### 3.1 CDs

**Figure 9:**
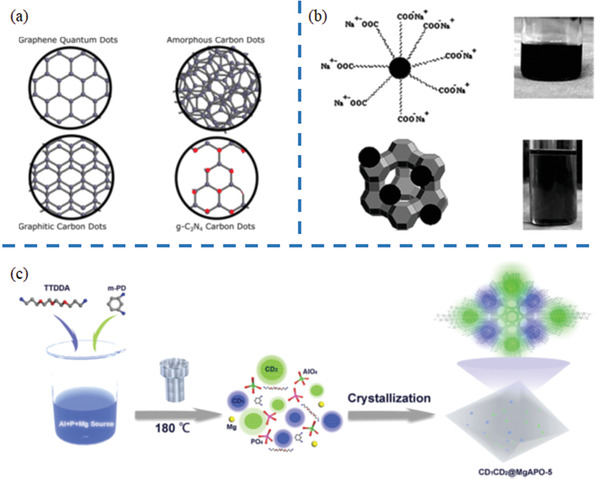
a) Schematic representation of commonly reported CD structures. Black and red dots represent carbon and nitrogen atoms, respectively. Reproduced under the terms of the CC‐BY Creative Commons Attribution 4.0 International license (https://creativecommons.org/licenses/by/4.0/).^[^ ^17^ ^]^ Copyright 2017, The Authors, published by MDPI. b) Synthetic methods of dispersed CDs and supported CDs; Reproduced with permission.^[^ ^155^ ^]^ Copyright 2008, American Chemical Society. c) Representative schematics of simultaneous formation of two kinds of CDs in one kind of zeolites. Reproduced with permission.^[^ ^172^ ^]^ Copyright 2019, The Royal Society of Chemistry.

Quantum dots (QDs) are a novel class of nanomaterials with 0D structures, and have gained significant recognition owing to their exceptional properties.^[^ ^166^, ^167^, ^168^, ^169^, ^170^ ^]^ Carbon dots (CDs) are defined as 0D and quasi‐spherical nanoparticles with sizes of less than 10 nm. In comparison with conventional semiconductor quantum dots, CDs have inspired intensive attention due to their unique advantages, such as excellent photostability, biocompatibility, low toxicity, low cost, simple synthesis, water solubility, chemical inertness, and so on, which afford CDs a variety of potential applications in biological, chemical, and optoelectronic fields. A classification of CDs was proposed based on their different core structures and morphologies including graphene quantum dots, graphitic carbon dots, amorphous carbon dots, and g‐C_3_N_4_ carbon dots^[^ ^171^ ^]^ (**Figure** **9**a). Zeolite templates can enable CDs into a variety of size, morphology, and chemical composition. Herein, CDs templated by zeolites are summarized.

The first example of CD templated by zeolites can be traced to 2008. As shown in Figure 9b, Giannelis et al. demonstrated the preparation of CDs templated by zeolites as the matrix.^[^ ^155^ ^]^ In this example, NaY (FAU) zeolite was selected as the matrix and 2,4‐diaminophenal dihydrochloride was employed as carbon source impregnated by ion‐exchanged method. After thermal oxidation, the CDs (≈4–6 nm) were grafted onto the external surface of NaY zeolite. Two years later, Chen et al. reported CDs confined in inner space of MAPO‐44 (CHA) zeolite via direct pyrolysis of the occluded cyclohexylamine template.^[^ ^157^ ^]^ Similar results are also achieved by loading additional organic molecules (acetone, ethanol, acetic acid) as carbon source. In recent years, there has been an increasing amount of literature on preparation of CDs@zeolite composites by the pyrolysis of organo‐templated zeolites.^[^ ^173^, ^174^ ^]^ Li et al. conducted a series of trials in synthetic methods of CDs@zeolite composites from aspects of modulation of the zeolite host matrix and organic templates and the calcination conditions (e.g., temperature and time).^[^ ^141^, ^172^, ^173^, ^174^, ^175^, ^176^, ^177^, ^178^, ^179^ ^]^ In their recent investigations,^[^ ^172^ ^]^ two kinds of templates were employed simultaneously in one synthetic system of zeolite. Interestingly, two kinds of CDs were obtained embedded into the pores of the zeolite (Figure 9c). Graphitic‐like C‐dots with diameter of 1.0–4.0 nm are generated in the channels of the zeolites. Garcia et al. carried out a number of investigations into the generation of CDs templated by small pore zeolites, for instance, ITQ‐29 (LTA) and ITQ‐12 (ITW); medium pore zeolites, for instance, silicalite (MFI) and large pore zeolites, for instance, pure silica Beta (BEA).^[^ ^159^ ^]^ CDs were rendered in the template of ITQ‐29 (LTA) and ITQ‐12 (ITW), while silicalite (MFI) and pure silica Beta (BEA) failed to produce CDs under the same condition.

To sum up, three key factors are ascribed to generation of CDs templated by zeolites: i) types of zeolite, ii) organic templates, and iii) carbon impregnation methods. The selection of proper zeolite matrix is a key for pyrolysis of organic templates in zeolites to generate CDs. Although it is not absolute, small pore zeolites might facilitate the generation of CDs in zeolites during heating treatment, whereas medium or large pore zeolites led the complete removal of occluded organic templates. To some extent, organic templates can determine the chemical composition of as‐prepared CDs. Different carbon impregnation methods result in variation in the location of CDs formation. CDs tends to form on the external surface of zeolites by the carbon impregnation method of ion exchange, while CDs should be embedded in the interrupted nanospaces of zeolites by pyrolysis of organic templates. Typically, the CDs formed on the external surface have a larger size (≈2–6 nm) and vice versa.

### 3.2 CNTs

**Figure 10:**
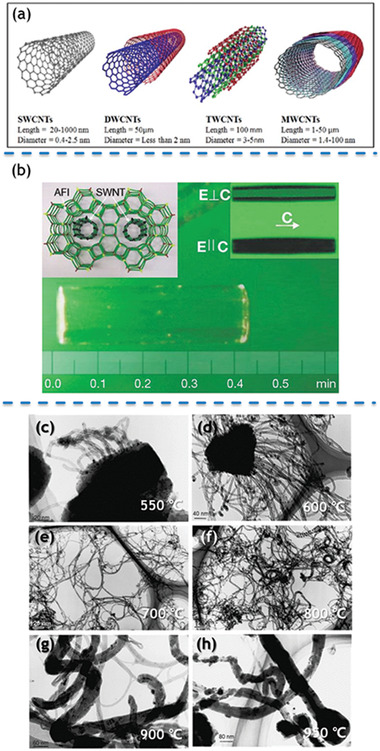
a) Types of carbon nanotubes; b) SWCNTs grown in 1D channels of AFI zeolite; c–h) TEM images of MWCNTs grown at different temperatures: c) 550 °C, d) 600 °C, e) 700 °C, f) 800 °C, g) 900 °C, h) 950 °C. Reproduced with permission.^[^ ^181^ ^]^ Copyright 2016, Ingenta. b) Reproduced with permission.^[^ ^64^ ^]^ Copyright 2000, Springer Nature. c–h) Reproduced with permission.^[^ ^182^ ^]^ Copyright 2004, Elsevier Ltd.

Typically, carbon nanotubes (CNTs) are tubes made of carbon with nanoscale diameters. After discovery by Iijima^[^ ^2^ ^]^ and Kiang et al.,^[^ ^180^ ^]^ continuous research related to CNTs emerged due to their fascinating physical and chemical properties. CNTs can be mainly categorized according to their diameter, lengths and the number of graphite sheets. As shown in **Figure** **10**a, four main types of CNTs are: i) single‐walled CNTs (SWCNTs), ii) dual‐walled CNTs (DWCNTs), iii) tri‐walled CNTs (TWCNTs), and iv) multi‐walled CNTs (MWCNTs).^[^ ^181^ ^]^ Ideally, SWCNTs are considered crystalline if the hexagonal aromatic structure of the carbon atoms has no variations in the graphene sheet. The diameter of SWCNTs ranges from 0.4 to 4 nm and the length varies from 20 to 1000 nm. As for MWCNTs, they can be visualized as more than three concentric cylindrical tubes of graphene sheets with diameter between 1.4 and 100 nm, and length between 1 and 50 µm, respectively. The general inter tubular distance in MWCNTs is ≈0.34 nm, which is consistent with the inter‐layered distance between two parallel graphene sheets in the graphite.

Zeolites with 1D nanochannels, for instance, AEL, AFI, LTL, MOR, etc. can shape carbon source into CNTs and prevent as‐prepared CNTs from agglomerating during their growth. The merits of zeolites as templates for CNTs are summarized as below:

1. Non‐continuous inner surface of zeolites can produce a homogenous dispersion of as‐prepared CNTs;
2. Homogeneously diffused catalytical sites contribute the synthesis of CNTs;
3. Ion exchange drastically increases the number of zeolites, which is also advantageous to the synthesis of CNTs;
4. Thermostability of zeolites enable formation of CNTs under high‐temperature treatment.

Hence, zeolites could be used as excellent supports to prepare CNTs.

In 2000, the world‐record smallest CNTs with a diameter of only 0.4 nm were discovered by our group.^[^ ^64^ ^]^ The CNTs were fabricated in the templates of zeolites, constituting an almost ideal one‐dimensional electronic system.^[^ ^6^ ^]^ The CNTs were prepared by the pyrolysis of tripropylamine molecules in the channels of porous zeolite AlPO4‐5 (AFI) single crystals. The structures of these single‐wall CNTs might fall into the zigzag (5,0) tube, which can be exactly capped by half a C_20_ fullerene (Figure 10b). Soon after that, the world record of smallest CNTs were refreshed by our group again.^[^ ^65^ ^]^ These single‐wall CNTs with diameter of 0.3 nm were fabricated in the elliptical nanochannels of an AlPO‐11 (AEL) single crystal. The structure of these CNTs was of (2,2) armchair symmetry, unambiguously revealed by polarized and resonant Raman scattering. Not only can the single‐wall CNTs be produced in the templates of zeolites, the multi‐wall CNTs can also be generated by the same method. Kumar et al. prepared the multi‐wall CNTs by thermal decomposition of a botanical hydrocarbon, camphor, on HY zeolite support (FAU) impregnated with Fe–Co binary catalyst.^[^ ^182^ ^]^ Influence of loading concentration of catalysts, temperature and vapor pressure of camphor were systematically investigated. In this example, multi‐wall CNTs with broad diameter (≈10 nm) were generated at lower temperature (600–700 °C), whereas single‐wall CNTs with narrow diameter (0.86–1.23 nm) were obtained at higher temperature (850–900 °C), as shown in Figure 10c–h. A series of functionalized CNTs templated by zeolites were fabricated via CVD method by Mokaya et al.^[^ ^63^ ^]^ In this case, ammonium‐exchanged zeolite‐*β* was employed as the template with ferric nitrate as the catalyst, and acetonitrile was selected as the carbon precursor. Mokaya et al. proposed that ammonium exchange of the zeolites play a vital role for the formation of aligned N‐doped CNTs, and the bamboo‐like morphology of the aligned N‐doped CNTs could be contributed to the hexagonal zeolite‐*β* template. The inner and outer diameters of the aligned N‐doped CNT were ≈10 and 30 nm, while the length of as‐prepared CNTs ranged from 2 to 10 µm depending on CVD temperature.

In brief, zeolites with 1D nanochannels, proper carbon precursor are necessary for preparation of zeolite templated CNTs. The morphology of the as‐prepared CNTs can be modulated by the reaction temperature and time. In addition, heteroatom doping could be also obtained by ion exchange and selecting different carbon precursors.

### 3.3 2D Graphene Nanosheets

Recently, 2D nanomaterials have been widely applied in electronics,^[^ ^183^, ^184^, ^185^, ^186^, ^187^, ^188^, ^189^ ^]^ photodetectors,^[^ ^190^, ^191^, ^192^, ^193^, ^194^, ^195^, ^196^, ^197^, ^198^, ^199^, ^200^, ^201^, ^202^, ^203^ ^]^ catalysis,^[^ ^204^ ^]^ biological medicine,^[^ ^205^, ^206^ ^]^ supercapacitor,^[^ ^207^ ^]^ and batteries^[^ ^208^ ^]^ because of their attractive photoelectric, electrochemical, and mechanical properties. Graphene, a 2D material in the form of a single layer of atoms with hexagonal lattice, is considered as an exciting material for its large theoretical specific surface area (2630 m^2^ g^−1^), excellent mechanical property, and remarkable electrical conductivity. For synthesis of graphene, there are top‐down methods such as micromechanical cleavage and liquid‐phase exfoliation, and bottom‐up methods such as CVD. Zeolites with laminar structures such as HEU, IWT, JNT, and MWW are all possible to serve as templates for synthesizing of 2D graphene. Nevertheless, there are relatively few examples by using zeolite as a template to synthesize 2D graphene.^[^ ^77^, ^91^, ^209^ ^]^

**Figure 11:**
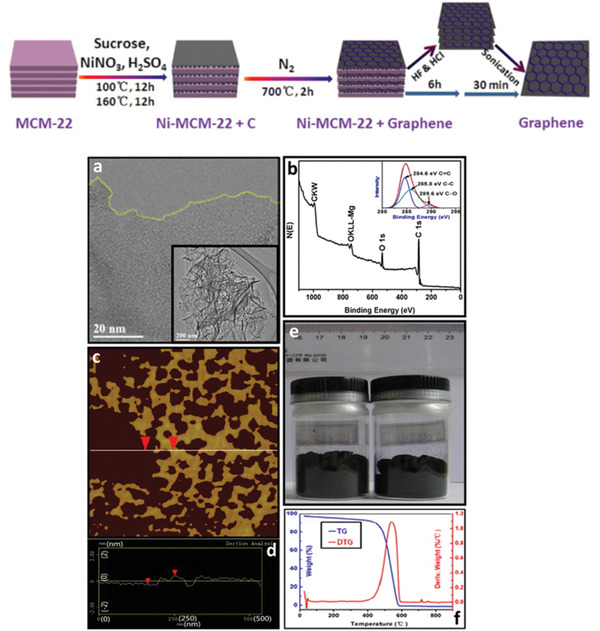
Synthetic process of graphene nanosheet by Ni‐MCM‐22 templates (top) and morphology, composition, thickness, yield, and thermostability of graphene nanosheets (bottom): a) TEM images of the graphene nanosheet; b) XPS of the graphene nanosheet; c,d) AFM image and thickness distribution of the graphene nanosheet; e) photograph of the samples of graphene nanosheet; f) thermogravimetry analysis of the graphene nanosheet. Top scheme and (a–f): Reproduced with permission.^[^ ^77^ ^]^ Copyright 2012, Elsevier Ltd.

One of the examples was reported by Kong et al., as shown in **Figure** **11**. Herein, MCM‐22 was selected as template due to its laminar structure. Sucrose and nickel cations can be introduced into the electronegative and porous frameworks of MCM‐22. In this case, nickel cations (Ni^2+^) served as catalysts and growth of 2D graphene was controlled in confined space of 2D pores. Similarly, graphene templated by MCM‐22 was also reported by Wang et al. for supporting ultrafine Pd nanoparticles. As‐prepared Pd‐supported nanocarbon materials not only inherited the nanosheet structure of pores in MCM‐22 zeolite with hierarchical structure, large surface area, and high conductivity, but also exhibited massive and accessible catalytic active sites. It is worth noting that high Al/Si ratio played a vital role for formation of optimal oxygen defects due to its Lewis acidity.

### 3.4 3D Porous Carbons

3D porous carbons are mostly investigated by academia and industry, since the single‐layer graphene framework without stacking is valuable to achieve a high surface area. As for 0D nanocarbon materials such as fullerene, the geometric surface area is 2625 m^2^ g^−1^. With regard to 1D nanocarbon materials such as single‐wall CNTs, the geometric surface areas depend on their diameters. For instance, single‐wall CNTs with 1 and 3 nm diameters have geometric surface areas of 1760 and 1460 m^2^ g^−1^, respectively. Toward 2D nanocarbon materials, the geometric surface area of graphene was calculated as high as 2627 m^2^ g^−1^. However, in reality, fullerenes tend to aggregate, while CNTs always bundle, and graphene could spontaneously stack. Hence, unfavorable surface loss inevitably occurs in low‐dimensional structures of nanocarbon materials. To reduce unfavorable surface loss, the concept of 3D porous graphene was proposed and the investigations on construction of 3D porous graphene has unprecedentedly flourished over the last few years.^[^ ^210^ ^]^ Despite their high surface areas, 3D porous graphene also possesses numerous unique properties: First, the high mechanical strength of graphene with large aspect ratio endow the porous frameworks of 3D graphene with enhanced stability. Hence, shrinkage or pore structure collapse, to some content, can be avoided. Second, 3D graphene can withstand harsh conditions, since graphene of these porous materials possess excellent thermal and chemical stability. Third, mass transfer can be accelerated by porous structure of 3D graphene. Meanwhile, the outstanding electrical conductivity of graphene is retained in the 3D graphene. Fourth, 3D graphene can be functionalized to graft numerous functional groups, which can serve as attractive supports for the loading of various organic or inorganic species. Porous features of 3D porous graphene can be distinguished into micro porosity, meso porosity, and macro porosity according to the definition of the International Union of Pure and Applied Chemistry (IUPAC), in which the micropores are defined as the pores with diameter smaller than 2 nm, mesopores are the pores with diameters between 2 and 50 nm, and macropores are defined as the pores larger than 50 nm. Therefore, 3D graphene materials increasingly appeal the attention from scientists especially on the realm of energy storage and conversion.

**Figure 12:**
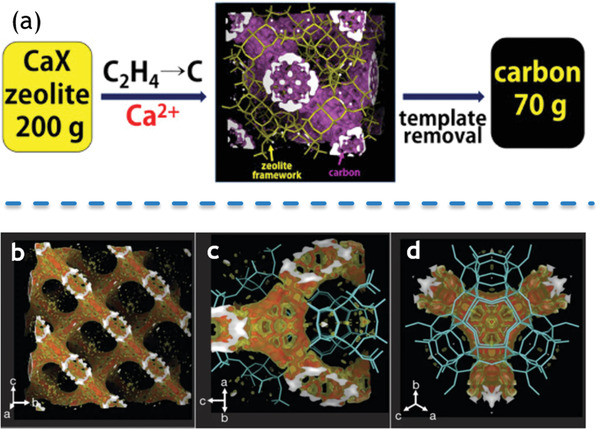
a) Synthetic process of 3D graphene‐like ordered microporous carbon templated by CaX zeolite. Reproduced with permission.^[^ ^20^ ^]^ Copyright 2017, Elsevier Ltd. b–d) 3D electron‐density map of the carbon framework templated by LaX zeolite. b–d) Reproduced with permission.^[^ ^21^ ^]^ Copyright 2019, Springer Nature Limited.

Zeolites are considered as ideal templates for the fabrication of various 3D porous graphene due to their thermostability, Lewis acidity, diversity of porous structures, etc. Nishihara et al. performed a series of experiments since 1990s to show that zeolite templates are enable to generate various 3D porous graphene.^[^ ^54^, ^56^ ^]^ The first attempt to employ zeolites as templates was reported by Kyotani et al. in 1997.^[^ ^60^ ^]^ In this case, Y zeolite (FAU) was selected as the template and poly(acrylonitrile) and poly(furfuryl alcohol) were introduced by polymer‐impregnation method, which were carbonized in the zeolite nanopores in the following process. In the same work, propylene gas as carbon source was introduced by CVD. It was found that the microscopic morphology of the resultant 3D porous carbons derived from the morphology and structure of the corresponding zeolite template. Similar investigations were simultaneously conducted by Mallouk et al.^[^ ^61^, ^140^ ^]^ Subsequently, Parmentier et al. conducted a series of trials in fabrication of porous nanocarbon materials using zeolite templates.^[^ ^211^, ^212^, ^213^, ^214^, ^215^ ^]^ Parmentier et al. focus on FAU‐type and EMT‐type zeolites as templates to prepare ordered microporous carbon material by the nanocasting process. Recently, an ordered microporous carbon material templated by EMC‐2 zeolite (EMT) was reported by Parmentier et al. Long‐range ordering in the material was revealed, which derived from the negative replication of the zeolite template. The structure of as‐prepared porous carbon was modeled by overlapped spherical voids, corresponding to the negative replication of EMC‐2 zeolite. Thisporous carbon material has a complex morphology due to its 3D interconnected porosity. Theoretically, the maximum of the pore size was calculated to 1.04 nm. In fact, the size of most pores was characterized with the pore size above 1.5 nm. Besides, Mokaya et al. also developed numerous nanocarbon materials templated by zeolites.^[^ ^63^, ^139^, ^216^, ^217^, ^218^, ^219^, ^220^, ^221^, ^222^, ^223^, ^224^, ^225^, ^226^, ^227^, ^228^, ^229^ ^]^ A series of novel methods were explored by Mokaya et al. to achieve higher surface area. A recent report from Mokaya et al. demonstrated the effects of zeolite compaction before use as templates.^[^ ^139^ ^]^ The pelletized 3D porous nanocarbon materials were prepared using compacted zeolite pellets, simultaneously exhibit higher porosity and higher packing density. When referring to zeolite‐templated 3D porous carbons, the investigations from Ryoo et al. cannot be ignored.^[^ ^20^, ^21^, ^81^, ^92^, ^99^, ^230^, ^231^, ^232^ ^]^ Since 1990s, Ryoo et al. synthesized and prepared a series of nanocarbon materials templated by zeolites. Massive efforts from Ryoo et al. were invested to elucidate the effect of cations of zeolites in synthesis and preparation of nanocarbon materials, as shown in **Figure** **12**. Specifically, Ryoo's laboratory selected zeolites with cations of alkaline earth and rare earth to develop a nanocarbon materials synthesis route.^[^ ^20^, ^21^ ^]^ Actually, high temperature is required for preparation of nanocarbon materials, which results in low selectivity. Introduction of lanthanide cations, which are excellent Lewis acid catalyst, can reduce temperature of the reaction and enhance the selectivity of the products. From such a simple idea, zeolites (BEA, EMT, and FAU) with cations of lanthanide were explored to successfully yield 3D microporous graphene with high selectivity. Besides, cation of Ca^2+^ was selected instead of La^3+^ for zeolite X with the purpose of low cost. A high carbon yield was achieved by ethylene carbonization, which is close to the case of zeolite with cation of La^3+^. As‐prepared nanocarbon materials possess 3D graphene structure and nanotube‐like frameworks along the inner surface of the zeolite with a small quantity of carbon deposition on the external surfaces. With cheaper template of Ca‐X and high yield of 70 g per batch, this synthesis route has demonstrated the potential for large‐amount production and practical applications.

## 4 Energy Applications of Nanocarbon Materials Templated by Zeolites

Nanocarbon materials templated by zeolites are widely employed for a variety of applications such as gas/vapor adsorption, catalysis, energy storage, biochemistry, and sensor.^[^ ^16^, ^54^, ^55^, ^56^ ^]^ Recently, their applications in energy storage and conversion have emerged such as fuel storage, electrocatalysis, and secondary battery. Combined with other nanocarbon materials, zeolite‐templated nanocarbons possess unique advantages for energy applications. Herein, recent advances in energy applications of zeolite‐templated nanocarbons are summarized from the following aspects of fuel storage, electrocatalysts, supercapacitor, and secondary battery. Some representative examples are listed in **Table** **1**.

**Table 1:** Representative examples of supercapacitors and rechargeable batteries based on zeolite‐templated nanocarbons

Supercapacitor				
Material^a)^	Morphology^b)^	Electrolyte	Specific capacitance	Reference
GM	Nanosheet	Aqueous KOH	233 F g^−1^	^[^ ^77^ ^]^
Quinone‐functionalized ZTC	Microporous carbon	Aqueous H_2_SO_4_	300–500 F g^−1^	^[^ ^100^ ^]^
Modified ZTC with oxygen‐functional groups	Microporous carbon	LiPF_6_/EC+DEC	330 F g^−1^	^[^ ^264^ ^]^

Rechargeable battery				
Material^a)^	Morphology^b)^	Application^c)^	Specific capacitance	Reference
Graphene Nanoribbons	Nanoribbon	LIB anode	1076 mAh g^−1^ at 0.2 C	^[^ ^272^ ^]^
NCO‐1	Graphitic carbon material with ordered microporous core and uniform mesoporous shell	LIB anode	1100 mAh g^−1^ at 0.1 C	^[^ ^98^ ^]^
FAU‐ZTC	Crystalline microporous carbon	AIB cathode	382 mAh g^−1^ at 0.05 C	^[^ ^82^ ^]^
FAU‐ZTC	Crystalline microporous carbon	MIB cathode	113 mA h g^−1^ at 0.1 C	^[^ ^273^ ^]^
FAU‐ZTC	Crystalline microporous carbon	DIB cathode	141 mA h g^−1^ at 0.12 C	^[^ ^80^ ^]^
A‐ZPC	Crystalline microporous carbon	Li‐S battery cathode	691 mA h g^−1^ at 1 C	^[^ ^276^ ^]^
ZTC/S‐20	Crystalline microporous carbon	Li‐S battery cathode	1199 mAh g^−1^ at 0.1 C	^[^ ^277^ ^]^

a) Material name reported in literature;

b) Morphology of the materials;

c) Application of the materials.

### 4.1 Gas Adsorbents

Porous materials such as zeolites, MOFs^[^ ^233^, ^234^, ^235^ ^]^ and COFs^[^ ^236^, ^237^, ^238^, ^239^ ^]^ are renowned for their gas adsorption. Compared with zeolites, MOFs and COFs, nanocarbon materials possess lower cost, larger specific surface area and lower density, which are beneficial to enhance gas adsorption.^[^ ^73^ ^]^ Nanocarbons templated by zeolites are advantageous for gas adsorption since they are distinguished from porous activated carbons with respect to their ordered pore framework and/or homogeneous pore size. Herein, some examples of nanocarbon materials for hydrogen storage and methane storage are demonstrated.

#### 4.1.1 Hydrogen Storage

With the development of industry and the improvement of human being's living standards, the demand for energy is also increasing. Since the energy used in recent decades are mainly came from fossil fuels (such as coal, oil and natural gas), which inevitably cause environment contamination, coupled with its limited reserves, it is extremely urgent to find renewable green energy. Hydrogen energy, as a kind of green energy and energy carrier with abundant reserves, wide sources and high energy density, is attracting widespread attention. The development and utilization of hydrogen energy has been highly valued by international society to expect of entering the "hydrogen economy" era in the middle of the twenty‐first century. Hydrogen energy utilization needs to solve the following three problems: hydrogen production, storage and application, and hydrogen energy storage and transportation is the key to hydrogen energy application.^[^ ^240^ ^]^ Generally, hydrogen exists in a gaseous form under standard conditions. Besides, it is flammable, explosive, and easy to diffuse. Therefore, in practical applications, safety, high efficiency, and no leakage loss in hydrogen storage and transportation are prior to solve.

Adsorption is a new hydrogen storage method that arose in recent years, which has plentiful advantages such as safety, reliability, and high storage efficiency. Among the materials for adsorbing hydrogen storage, carbonaceous materials are the best adsorbents, which are not only insensitive to a small number of gaseous impurities, but can possess excellent cycling stability. The carbonaceous materials for hydrogen storage are mainly activated carbon (AC) with high specific surface area, graphite nanofibers (GNFs), and carbon nanotubes (CNTs).

Nanocarbon materials templated by zeolites are more promising materials for hydrogen storage, since they possess higher specific surface area and their structure and morphology are easily tuned in comparison with AC, GNFs and CNTs. There are three widely used approaches to enhance the capacity of hydrogen storage: 1) specific surface area improvement, 2) loading of noble metal nanoparticles, 3) employment of borohydride‐encapsulated nanocarbons.

**Figure 13:**
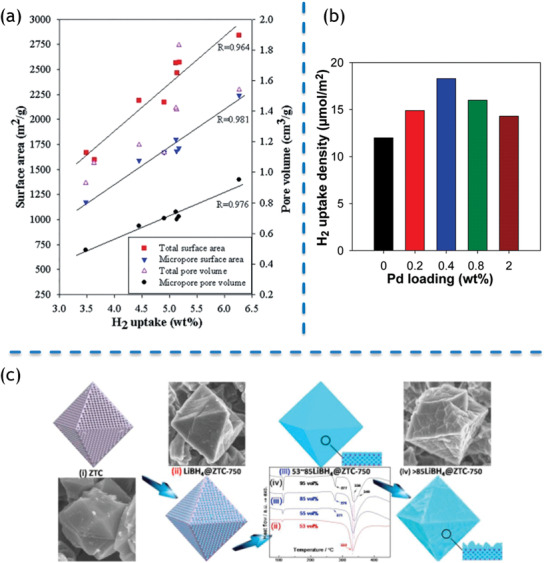
a) Correlation between H_2_ uptake and surface area/pore volume. Reproduced with permission.^[^ ^247^ ^]^ Copyright 2016, John Wiley & Sons. b) Correlation between H_2_ uptake and loading of noble metal nanoparticles. Reproduced with permission.^[^ ^218^ ^]^ Copyright 2013, American Chemical Society. c) LiBH_4_‐loaded zeolite‐templated porous carbons for hydrogen storage. Reproduced with permission.^[^ ^248^ ^]^ Copyright 2015, Elsevier B.V.

The relation between specific surface area of the nanocarbons templated by zeolites and hydrogen adsorption amount has already been thoroughly investigated.^[^ ^79^, ^227^, ^241^, ^242^, ^243^, ^244^, ^245^, ^246^, ^247^ ^]^ Moreover, it has been revealed that H_2_ uptake also depends on pore volume, specifically micropore volume. Xia et al. systematically investigated the correlation between H_2_ uptake and specific surface area as well as pore volume.^[^ ^247^ ^]^ In this case, Xia et al. employed a series of synthesis strategies to prepare the porous carbon materials templated by zeolites with variable structural ordering and tunable textural properties, which possess surface areas of 1600–2850 m^2^ g^−1^, pore volumes of 1.0–1.8 cm^3^ g^−1^, and exhibit hydrogen‐uptake capacities in the range of 3.4–6.3 wt% (at −196 °C and 20 bar). It is worth noting that linear relationships were obtained between hydrogen‐uptake capacity and the specific surface area. The similar correlations were also found between hydrogen‐uptake capacity and pore volume, suggesting that specific surface area and pore volume play a significant role in the hydrogen adsorption (**Figure** **13**a).

Loading noble metal nanoparticles is an efficient approach to enhance H_2_ uptake by chemisorption.^[^ ^249^, ^250^, ^251^ ^]^ Zeolite‐templated nanocarbon materials doped with noble metal nanoparticles have been widely explored to improve H_2_ uptake.^[^ ^218^, ^223^, ^252^, ^253^ ^]^ The process of chemisorption in zeolite‐templated nanocarbons can be described as below: H_2_ molecules are dissociative adsorbed on the surface of noble metal particles. Subsequently, atomic hydrogens (H radicals) migrate to the zeolite‐templated nanocarbon matrix. Mokaya et al. developed a supercritical fluid (SCF) mediated metal incorporation approach to deposit palladium nanoparticles onto zeolite‐templated nanocarbon matrix. As prepared samples are porous nanocarbons decorated with well‐dispersed Pd nanoparticles, exhibiting enhanced hydrogen storage performance. Although doping Pd lowered the specific surface area, Pd‐doped nanocarbons enhanced the H_2_ uptake (14.3–18.3 µmol H_2_ m^−2^) in comparison with that of Pd‐free nanocarbons (12.0 µmol H_2_ m^−2^). Thus, it is essential to deposit Pd nanoparticles onto the nanocarbons for the enhancement of hydrogen uptake and optimize the balance between loading amount of metal particles and surface area to achieve the optimal nanocomposites for hydrogen storage (Figure 13b).

Porous nanocarbons is also suitable to load borohydrides for hydrogen storage.^[^ ^248^ ^]^ Chen et al. provided a new strategy to uptake and release H_2_ close to the target temperature (233–333 K), as shown in Figure 13c. Herein, LiBH_4_ was confined into zeolite‐templated nanocarbons with high porosity and excellent mechanical stability. As‐prepared nanocomposites can release H_2_ at 194 °C, much lower than that of the bulk LiBH_4_ (375 °C). The activation energy of hydrogen desorption is dramatically reduced by 60.4 kJ mol^−1^. Meanwhile, the foaming effect of desorption almost eliminated.

#### 4.1.2 Methane Storage

Natural gas, of which the main composition is methane, is a natural resource with abundant storage, low pollution, and low cost. It is an ideal alternative for traditional fuels such as coal and petroleum. However, the low energy density of natural gas (only 0.11% of gasoline) and the low critical temperature (*T*_c_ = 191 K) limit its practical applications. It has become an important research topic to increase the energy density of natural gas storage. Adsorption of natural gas (ANG) technology is a promising natural gas storage technology. Compared with compressed natural gas widely used in industry, it possesses numerous merits such as low gas storage pressure (3.5–6.0 MPa), simple gas cylinder preparation process, high safety, and cheap maintenance costs.

**Figure 14:**
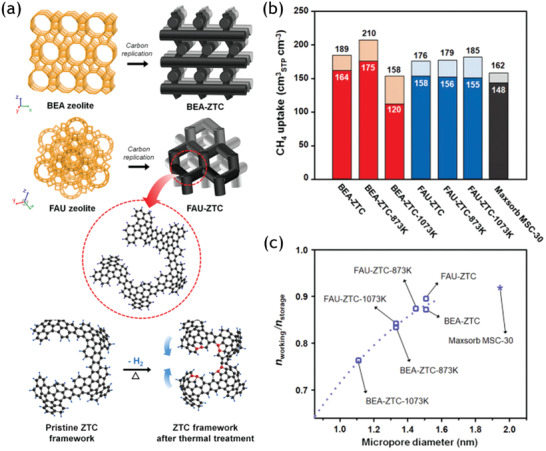
a) Synthetic process of zeolite‐templated carbons with further dehydrogenation and densification; b) volumetric CH_4_ storage capacities (light color) and working capacities (deep color) of as‐prepared zeolite‐templated carbons; c) the ratio between the working capacity and storage capacity as a function of carbon micropore diameter. a–c) Reproduced with permission.^[^ ^254^ ^]^ Copyright 2018, Elsevier Ltd.

Zeolite‐templated nanocarbons are also investigated to apply in the area of methane storage^[^ ^255^, ^256^, ^257^, ^258^, ^259^, ^260^ ^]^ aiming to develop their practical applications in vehicle industry. Similar to hydrogen storage, some approaches have been employed to improve methane uptake. Recently, Choi et al. indicated the influence of pore size on methane storage. In this case, two different zeolite structures, BEA and FAU were selected as template to prepare porous nanocarbons.^[^ ^254^ ^]^ Various pore structure of zeolite‐templated nanocarbons are obtained by using different zeolite templates, as shown in **Figure** **14**. Meanwhile, the pore sizes can be tuned by thermal contraction with pore sizes of zeolite‐templated nanocarbons ranging from 1.1 to 1.5 nm. Among as‐prepared nanocarbons, the samples with micropores smaller than 1.3 nm showed dramatic increases in the isosteric heat of adsorption with increasing CH_4_ coverage, which strongly indicated the occurrence of substantial lateral interactions between the methane in relatively smaller pores. The sample prepared in BEA zeolite with subsequent treatment at 873 K showed the optimal methane storage capacity of 210 cm^3^ STP cm^−3^), which revealed that its optimum porous structures and sizes are essential to methane storage.

### 4.2 Supercapacitors

Supercapacitors, also known as electrochemical capacitors, are based on their high power density (5–30 kW kg^−1^, 10–100 times higher than lithium‐ion batteries), extremely short charging times (minutes or even tens of seconds), long cycle life (104–106 times) has received wide attention in the field of energy storage. According to the structure of devices and energy storage mechanism, supercapacitors can be divided into three categories: electric double layer capacitors (EDLCs), pseudocapacitors, and asymmetric supercapacitors. Asymmetric supercapacitors cover a wide range including capacitive asymmetric supercapacitors and hybrid capacitors. The hybrid capacitor is to introduce the battery characteristic material or the redox active electrolyte into the all‐capacitor device. Based on the Faraday redox process of these materials, the energy density of the hybrid capacitor is significantly improved compared with other types of supercapacitors, while retaining a high‐power density and cycle stability. Zeolite‐templated nanocarbons possess sufficient electrical conductivity and excellent microporosity, which endow them tremendous potential as electrode materials in supercapacitors.^[^ ^73^, ^75^, ^76^, ^77^, ^78^, ^79^, ^84^, ^85^, ^86^, ^89^, ^90^, ^99^, ^100^, ^142^, ^149^, ^261^, ^262^, ^263^, ^264^, ^265^, ^266^, ^267^ ^]^ Herein, zeolite‐templated nanocarbon‐based supercapacitors are summarized from the aspects of EDLCs, pseudocapacitors, and asymmetric supercapacitors.

**Figure 15:**
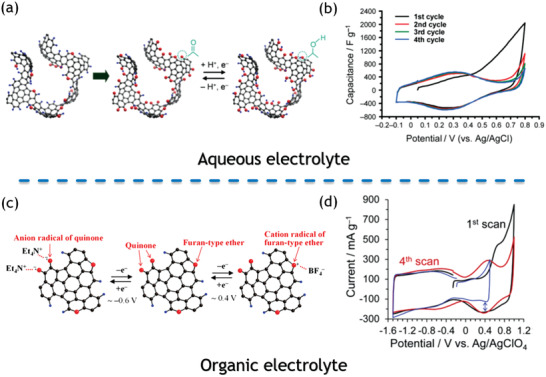
a) Charging and discharging process of quinone‐functionalized zeolite‐templated carbon in aqueous electrolyte. b) CV curves of quinone‐functionalized zeolite‐templated carbon measured with a three‐electrode system in 1 m H_2_SO_4_. c) Charging and discharging process of quinone‐functionalized zeolite‐templated carbon in organic electrolyte. d) CV curves of quinone‐functionalized zeolite‐templated carbon measured with a three‐electrode system in 1 m Et_4_NBF_4_/PC. a,b) Reproduced with permission.^[^ ^100^ ^]^ Copyright 2014, The Chemical Society of Japan. c,d) Reproduced with permission.^[^ ^264^ ^]^ Copyright 2015, Elsevier Ltd.

As for EDLCs, specific surface area of zeolite‐templated nanocarbons as high as 3800 m^2^ g^−1^ is necessary but not enough to improve specific capacitance since some segments of the materials are very narrow and exhibit semiconductor properties.^[^ ^142^, ^266^ ^]^ Compared to activated carbons, zeolite‐templated nanocarbons possess active edge sites, which are easily oxidized the anodic oxidation, thereby exhibiting a large pseudocapacitance. In aqueous and organic electrolytes, various oxygen‐functional groups can be introduced on the edge sites of zeolite‐templated nanocarbons. Nishihara et al. employed H_2_SO_4_ and Et_4_NBF_4_/PC as electrolytes to investigate pseudocapacitance of functionalized zeolite‐templated nanocarbons.^[^ ^100^, ^264^ ^]^ In the case of acidic electrolyte, the edge sites of zeolite‐templated nanocarbons are easily substituted by massive quinone groups through electrochemical oxidation. (**Figure** **15**a,b) The quinone‐functionalized nanocarbons exhibit a high pseudocapacitance (300–500 F g^−1^) around 0.28 V (vs Ag/AgCl) in an aqueous electrolyte solution (1 m H_2_SO_4_) even after thousands of charge‐discharge cycles because of the reversible quinone‐hydroquinone redox reactions.^[^ ^100^ ^]^ With regard to organic electrolyte, tetraethylammonium tetrafluoroborate/propylene carbonate (Et_4_NBF_4_/PC), a large amount of redox‐active oxygen‐functional groups are introduced by this anodic oxidation process, in which PC is employed as an oxygen source, as shown in Figure 15c,d. Oxygen‐functionalized nanocarbons exhibit a high specific capacitance (330 F g^−1^) in organic electrolyte.^[^ ^264^ ^]^ The large pseudocapacitance at two potential regions are considered to originate from the formation of anion and cation radicals of quinones and ethers, respectively. Asymmetric capacitors were investigated to broaden potential window and improve the energy density. Recent study from Nishihara et al. shows that zeolite‐templated nanocarbons and KOH‐activated carbon were selected as a pseudocapacitive positive electrode and a stable negative electrode to construct asymmetric capacitor. The asymmetric capacitor featured a wide potential window and exhibited a high energy density of 24.5 W h kg^−1^.

### 4.3 Secondary Battery

A secondary battery, also called a rechargeable battery or a battery, referring to a battery which can be recovered by activation of the active materials after discharge. By employing the reversibility of chemical reactions, a new strategy to assemble battery can be built up: after a chemical reaction is converted into electrical energy, the chemical system can be repaired with electrical energy, and then converted into electrical energy by chemical reaction, so it is called a secondary battery or rechargeable battery. The main rechargeable batteries on the market are nickel metal hydride battery, nickel‐cadmium battery, lead‐acid battery, lithium ion battery, etc.^[^ ^268^ ^]^ In academia, rechargeable batteries such as sodium‐ion batteries, metal‐air batteries, lithium‐sulfur batteries, and flow batteries also attract intense attention from global context due to their light weight, small self‐discharge, high energy density, and environmental friendliness.

There have already been many reports that zeolite‐templated nanocarbons were employed as secondary battery electrode due to their large surface area, relatively high conductivity, tunability of structures, and excellent capability to immobile active materials. Herein, recent developments of zeolite‐templated nanocarbon for rechargeable battery electrodes are summarized.

#### 4.3.1 Lithium‐Ion Battery

Lithium‐ion battery (LIB) is a rechargeable battery that relies on lithium ions to move between the cathodes and anodes to work. Embedded lithium compounds are employed in lithium‐ion batteries as an electrode material. “For the development of lithium‐ion batteries,” John B. Goodenough was awarded the 2019 Nobel Prize in chemistry, jointly with Stanley Whittingham and Akira Yoshino.

Currently, anode materials are broadly classified into carbon materials, silicon‐based and their composite materials, nitride anodes, tin‐based materials, lithium titanate, alloy materials, etc.^[^ ^269^ ^]^ Among them, carbon materials are the mainstream of anode of LIB.^[^ ^270^, ^271^ ^]^ Moreover, most of the commercial lithium batteries adopt carbon materials as anode. The reason why carbon materials were selected as anode are listed as below: First, the potential of carbon materials is low, the discharge platform is approximately from 0.01 to 0.2 V, which easily enable as‐assembled devices to obtain a higher output voltage. Second, the layered stacking structure of carbon materials allows lithium ions to freely shuttle between layers with less hinder. Finally, the carbon abundance is rich reserves, easy to be obtained directly.

**Figure 16:**
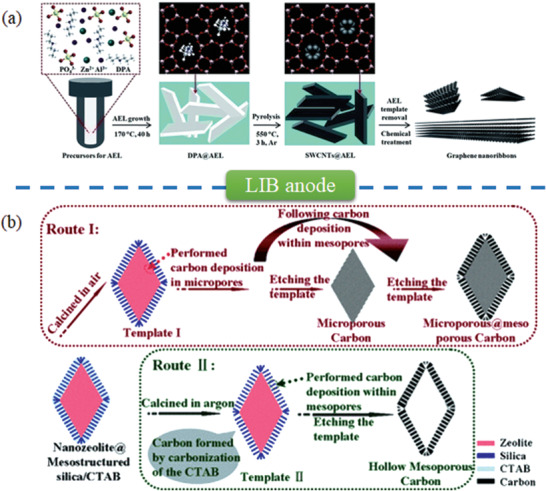
a) Graphene nanoribbons from zeolite‐templated CNTs for lithium ion storage. Reproduced with permission.^[^ ^272^ ^]^ Copyright 2018, The Royal Society of Chemistry. b) A core–shell‐structured graphitic carbon material by using a nanozeolite@mesostructured silica composite molecular sieve as the template for lithium‐ion storage. Reproduced with permission.^[^ ^98^ ^]^ Copyright 2013, Wiley‐VCH.

There have been some reports on nanocarbon materials templated by zeolite as anode materials for LIBs. Recently, Ruan et al. collaborated with our group developed a kind of graphene nanoribbon templated by zeolite as anode of LIBs.^[^ ^272^ ^]^ Previously, the armchair (2,2) carbon nanotubes templated by zeolite ZnAPO4‐11 (AEL) was reported by our group.^[^ ^65^ ^]^ By removing the zeolite template, the armchair carbon nanotubes can transform into N‐doped graphene nanoribbon. The thickness of as‐prepared graphene nanoribbon ranged from two to seven layers, the width ranged from 10 to 30 nm and length exceeded 1 µm. The as‐prepared graphene nanoribbons exhibit high performance as anode materials for LIBs, as shown in **Figure** **16**a. The high capacities of the as‐assembled anodes could be attributed to following reasons: 1) N‐doping increased electrical conductivity and electrochemical activity of graphene; 2) a high specific surface area enable fast charge transfer, enhanced charge capacity, and high adsorption of lithium ion; 3) topological and chemical defects in graphene nanoribbon improve the faradaic capacitance, thereby promoting ion diffusion.

Zhao et al. developed a series of hierarchical meso‐/micro‐porous nanocarbons templated by both zeolites and mesoporous silicas.^[^ ^97^, ^98^ ^]^ A new core‐shell‐structured nanocarbon materials with both micropores and mesopores was prepared by full carbon deposition in the template, exhibiting high reversible capacity, excellent rate capacity, and long cyclic stability, as shown in Figure 16b. Hierarchical structures of nanocarbon materials have unique advantages as anode materials for LIBs: 1) Large surface area of microporous nanocores enable high‐capacity storage of lithium ions; 2) highly opened mesopores of mesoporous shells enhance lithium ions and electrons transfer into inner cores.

#### 4.3.2 The Aluminum Battery, the Magnesium‐Ion Battery, and Dual‐Ion Batteries

Recently, Stadie et al. extend the applications of zeolite‐templated nanocarbon materials to the aluminum battery (AB),^[^ ^82^ ^]^ the magnesium‐ion battery,^[^ ^273^ ^]^ and dual‐ion batteries.^[^ ^80^ ^]^

##### Aluminum Battery

Practical rechargeable batteries with Al anodes were reported until recently because of the formation of solid‐electrolyte interphase (SEI) and lagged development of suitable electrolytes.^[^ ^274^, ^275^ ^]^ Graphite‐based materials are employed as cathode with ionic liquid of [EMIm]Cl as electrolyte, which can mitigate and avoid the interphase formation. Nevertheless, the larger size of the AlCl_4_^−^ ions hinders intercalation and unfavorable oxidation of graphite exists in discharging process. Via the introduction of macroscopic voids by engineering of the graphite morphology is an efficient approach to remedy low rate capacity. Nanocarbon materials templated by zeolites possess high specific surface area and conductive framework with tailored porosity can precisely accommodate the various ion species.

**Figure 17:**
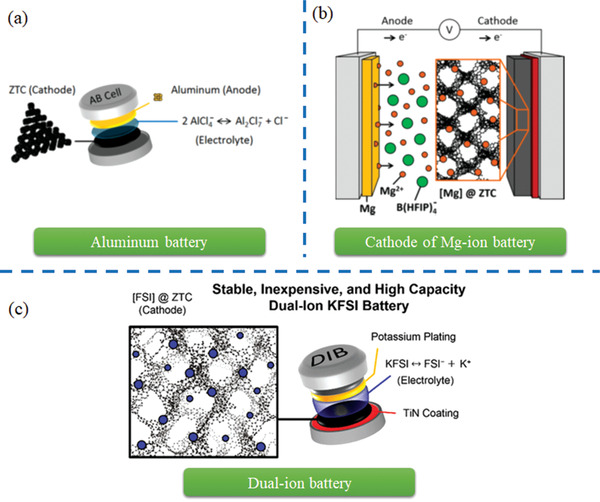
a) Zeolite‐templated nanocarbon for aluminum battery. Reproduced with permission.^[^ ^82^ ^]^ Copyright 2017, American Chemical Society. b) Zeolite‐templated nanocarbon for cathode of magnesium battery. Reproduced with permission.^[^ ^273^ ^]^ Copyright 2019, American Chemical Society. c) Zeolite‐templated nanocarbon for dual‐ion batteries. Reproduced with permission.^[^ ^80^ ^]^ Copyright 2019, American Chemical Society.

Nanocarbons templated by faujasite (FAU) with pore of ≈1.2 nm is suitable for the fast and dense storage of AlCl_4_^−^ ions with radius of 0.6 nm.^[^ ^82^ ^]^ As‐assembled battery showed high specific gravimetric and volumetric energy up to 64 Wh kg^−1^ and 30 Wh L^−1^. The corresponding specific gravimetric and volumetric powers were up to 290 W kg^−1^, 93 W L^−1^. A good reversibility was implemented within the potential ranged from 0.01 to 2.20 V with high cycling stability (**Figure** **17**a).

##### Magnesium‐Ion Battery

The same sample, faujasite‐templated porous carbon was subsequently applied as cathode for Mg‐ion batteries (MIBs), which are fascinating for their high theoretical anodic capacity of 3833 mA h mL^−1^. Recent developments of Mg‐ion electrolyte improved voltage of MIBs, however boosted the requirement of cathode materials. It is necessary to tailor proper porosity for diffusion and storage of Mg ion; moreover, to decrease the interaction between the host and the intercalant is essential to improve the kinetics of Mg‐ion storage at the cathode.

Stadie et al. explored a Mg‐ion cathode assembled by porous carbon templated by FAU zeolite.^[^ ^273^ ^]^ FAU zeolite‐templated porous carbon possess ordered porosity and high conductivity. As‐assembled devices exhibited high of 113 mA h g^−1^ after 100 cycles with an average discharge voltage of 1.44 V. Relatively high specific capacities were retained of 76% after 200 cycles (Figure 17b).

##### Dual‐Ion Batteries

The mechanism of the dual‐ion battery (DIB) differs from the conventional LIB: during the charging process, the cathode undergoes an anion intercalation reaction, while the anode undergoes an aluminum‐lithium alloying reaction, and the discharge process is reversed. Development of dual‐ion battery not only significantly increases the operating voltage of the battery (3.8–4.6 V), but also greatly reduces the weight, volume, and manufacturing cost of the battery, thereby comprehensively increasing the energy density and lifespan of the battery.

In the application of dual‐ion battery, the capacity of anion storage in zeolite‐template nanocarbon materials was explored. In the example of dual‐ion battery reported by Stadie et al., bis(fluorosulfonyl)imide (FSI^−^) was selected as an optimal anion due to its reported successful utilization in DIBs.^[^ ^80^ ^]^ Potassium was selected as a suitable cation due to its high natural abundance and excellent electroplating/stripping properties. The most well‐understood FAU zeolite‐templated porous carbon was employed as cathode due to its high specific surface area, ordered porous structure, and applicable size of pores. Relatively high potential of zeolite‐templated carbon cathode of as‐assembled battery versus K/K^+^ result in both high specific energy (up to 176 Wh kg^−1^, 79.8 Wh L^−1^) and high specific power (up to 3945 W kg^−1^, 1095 W L^−1^), while the selection of FSI‐electrolyte mitigate the formation of SEI and increase the cycling stability (Figure 17c).

#### 4.3.3 Lithium–Sulfur Batteries

Zeolite‐templated nanocarbon materials have also been applied as cathode of the lithium–sulfur battery.^[^ ^276^, ^277^ ^]^ Lithium–sulfur (Li–S) batteries have become one of the most promising alternatives of LIBs for next‐generation energy storage devices due to its overwhelming theoretical energy density (≈2600 Wh kg^−1^), low cost, and environmental friendliness. Currently, there are obvious shortcomings. Thus, the distance from mass production is relatively far. Herein, only for main drawbacks are summarized: 1) Li anodes are inclined to react with electrolyte. Hence, it is easy to form SEI with high resistivity and low Coulomb efficiency; 2) the product of the discharge process, Li_2_S, is insulated, generating irreversible components in the reaction process and poor cycling performance; 3) slight changes in the assembly of Li electrode will lead to changes in the overall performance of the battery, and there are many uncontrollable factors; 4) the “shuttle effect” exists and self‐discharge rate is very large.

**Figure 18:**
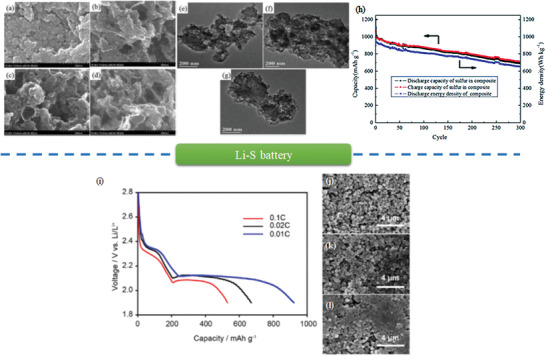
a–d) SEM images of NaX zeolite (a), zeolite‐templated porous carbon (b), amphiphilic zeolite‐templated porous carbon (c), and amphiphilic zeolite‐templated porous carbon with S loaded in the pores (d). e–g) TEM images of zeolite‐templated porous carbon (e), amphiphilic zeolite‐templated porous carbon (f), amphiphilic zeolite‐templated porous carbon with S loaded in the pores (g), and h) the discharge energy density of amphiphilic zeolite‐templated porous carbon. a–h) Reproduced with permission.^[^ ^276^ ^]^ Copyright 2016, The Royal Society of Chemistry. i) Discharge profiles for zeolite‐templated nanocarbon with particle size of 400 nm as cathode. j–l) SEM images of the zeolite‐templated nanocarbon samples after discharge at different current rates: j) 0.1C, k) 0.02C, and l) 0.01C. i–l) Reproduced with permission.^[^ ^277^ ^]^ Copyright 2018, Wiley‐VCH.

To overcome the drawbacks of cathode of Li–S batteries, it is essential to develop conductive materials with better sulfur‐carbon contact and polysulfide retention. Wu et al. modified conventional zeolite‐templated porous carbon for cathode of Li–S batteries. Na‐X was selected as template to prepare porous carbon with high conductivity. Besides, *N*‐polyvinylpyrrolidone (PVP) was employed to modified the amphiphilicity of as‐prepared porous carbon, which can load 46 wt% of sulfur. The voltage range of as‐assembled battery is 1.7–2.6 V by using the electrolyte of 1.0 m lithium bis(trifuoromethanesulfonyl)imide (LiTFSI) and 0.1 m LiNO_3_ dissolved in 1,3‐dioxolane (DOL) and 1,2‐dimethoxyethane (DME) (1:1, v/v). The energy capacity of 670 mA h g^−1^ was achieved with high cycling stability (**Figure** **18**a–h).

In the discharging process of the Li–S battery, pore blockage by discharge products lead to low utilization of active materials. To solve this problem, Choi et al. prepared a series of microporous carbons with various particle sizes from 20 to 400 nm, but identical micropore size of 1.4 nm, to investigate the particle size effect for cathodes of Li–S batteries.^[^ ^277^ ^]^ The sample with smallest particle size exhibit optimal capacity of 1199 mAh  g^−1^ and high cycling stability. For the case of microporous carbons with larger particle sizes, Li_2_S was generated at the entrance of the micropores, thereby limiting discharging capacity. Thus, both pore sizes and particle sizes are crucial factors in rational design of cathode materials for the Li–S battery (Figure 18i–l).

#### 4.3.4 All‐Organic Proton Batteries

All‐organic proton battery is a special class of rechargeable batteries that use organic matter as electrode materials, which are clean, sustainable and inexpensive alternatives of metal‐containing energy storage devices.^[^ ^278^ ^]^ However, organic matter tends to dissolve in electrolyte, thereby restricting the development of all‐organic proton battery. Microporous carbons have been considered as attractive candidates to immobilize active materials in the electrode of all‐organic proton battery. Zeolite‐templated microporous carbons possess numerous unique properties, such as regularity of pores, diversity of structures, and plentiful edges of graphene.

Regularity of pores, diversity of structures of zeolite‐templated porous carbon ensure the accommodation of active materials, while plentiful edge sites can be functionalized by active materials.^[^ ^85^ ^]^ Nishihara et al. employ zeolite Y to prepare 3D framework of nanographene, which possess plentiful edge sites of graphene. As‐prepared microporous carbon is a suitable host for quinones as anode of all‐organic proton battery.^[^ ^100^ ^]^ Quinone‐functionalized microporous carbon possesses a high pseudocapacitance derived from quinone/hydroquinone redox couple. Moreover, loading amount of quinones is calculated up to 60 wt%, thereby exhibiting enormous potential as anode of all‐organic battery. Subsequently, Honma et al. adopted this strategy to assemble all‐organic proton batteries.^[^ ^83^ ^]^ Active materials, anthraquinone and tetrachlorohydroquinone were selected to be loaded in the zeolite‐templated nanocarbons by impregnation method. In discharging process, anthraquinone was oxidized as proton acceptor, while tetrachlorohydroquinone was reduced as proton donor. Limited space of pores prevents quinone derivatives from crystallization. The capacity of as‐assembled devices is as high as 30.6 W h kg^−1^, comparable with that of reported conductive polymer host.

#### 4.3.5 Fuel Cell

A fuel cell is a power generation device, which converts chemical energy in a fuel into electrical energy by performing a redox reaction mainly by oxygen or other oxidants. There are many types of fuel cells. Among them, solid oxide fuel cell (SOFC), direct methanol fuel cell (DMFC) and proton‐exchange membrane fuel cell (PEMFC) will be the most promising technical routes.

The methanol oxidation reaction (MOR) is a key technique for DMFC. Pt‐loaded nanocarbon materials have been reported with excellent electrocatalytical activity for MOR. Zeolite‐templated porous carbons is promising platform to loading Pt nanoparticles.^[^ ^88^, ^94^, ^279^ ^]^ Liu et al. reported porous carbon templated by various hard templates to anchor Pt nanoparticles derived from H_2_PtCl_6_. The porosity of as‐prepared nanocomposites is various including microporosity, mesoporosity, and macroporosity. Pt nanoparticles are highly diffused in the porous carbons, which have tremendous contribution to MOR activity. MOR activity is strongly associated with pore size of porous carbon support: the superior MOR performance was demonstrated by microporous carbon support. However, the nanocomposites with micropores exhibit improved tolerance for CO poisoning than those with mesopores and macropores.

Pt‐loaded nanocarbon materials templated by zeolites have also been applied in cathode materials of PEMFC for oxygen reduction reaction (ORR).^[^ ^93^, ^95^, ^96^ ^]^ One of the examples from Eric N.Coker el al. present a method for the preparation of size‐controlled Pt clusters on nanostructured carbon with tunable porosity.

The process included four main steps: 1) Pt clusters were introduced in zeolites; 2) carbon precursors were impregnated in zeolites; 3) pyrolysis; 4) the zeolites were removed. Structure and morphology of as‐prepared nanocomposites can be controlled by the precise synthesis conditions. The microporous carbons with Pt nanoparticles with sizes from 1.3 to 1.7 nm exhibited excellent ORR performance, which is comparable with commercial Pt/C electrode.

### 4.4 Eletrocatalysis

The applications of zeolite‐templated nanocarbon materials in electrocatalysis focus on oxygen reduction reaction (ORR),^[^ ^81^, ^92^, ^138^, ^142^, ^230^ ^]^ which is a very important part of the energy conversion reaction and has important applications in fuel cells, metal–air batteries, and electrolytic cells. Currently, the best ORR catalysts are Pt‐based catalysts, especially Pt based alloy catalysts such as PtNi. In addition, the systems of catalysts are mostly investigated including Fe–N–C, Ni–N–C and doped graphene‐based materials.

**Figure 19:**
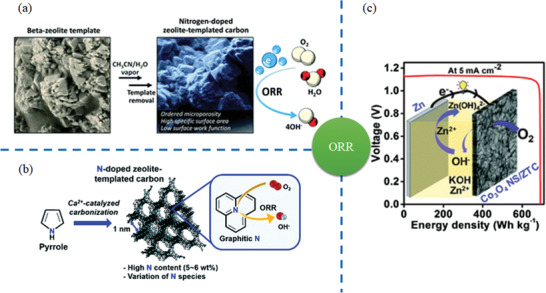
a) N‐doped zeolite‐templated carbon as a metal‐free electrocatalyst for oxygen reduction. Reproduced with permission.^[^ ^92^ ^]^ Copyright 2016, The Royal Society of Chemistry. b) The effect of variation of nitrogen species in zeolite‐templated carbon on oxygen reduction activity. Reproduced with permission.^[^ ^230^ ^]^ Copyright 2019, The Royal Society of Chemistry. c) Co_3_O_4_ nanosheets on zeolite‐templated carbon as an efficient oxygen reduction‐Reproduced with permission.^[^ ^81^ ^]^ Copyright 2019, The Royal Society of Chemistry.

ORR catalysts based on zeolite‐template nanocarbons are mostly investigated by Ryoo et al.^[^ ^81^, ^92^, ^230^ ^]^ Recently, N‐doped zeolite‐templated nanocarbons were systematically studied especially in the effect of pyridinic, pyrrolic, and graphitic N species, as shown in **Figure** **19**a.^[^ ^92^ ^]^ Ca^2+^‐exchanged Y zeolite was employed as a template to lower the carbonization temperature, which make it possible to modulate N‐containing species by varying reaction temperature. As‐prepared nanocarbons possess similar porous structure with diverse N‐containing groups, which is a suitable platform to investigate the effect of N‐containing groups without influence of porosity. The results indicate the graphitic N was most effective groups for ORR performance among the three N‐containing groups, as shown in Figure 19b.^[^ ^230^ ^]^ Transition metal oxide‐based nanocarbons were also reported by Ryoo et al. Hydrophobic zeolite‐templated nanocarbon was employed as substrate to prepare Co_3_O_4_ nanosheets, as shown in Figure 19c.^[^ ^81^ ^]^ Borohydrides were selected as reductants followed by oxidation with oxygen to generate oxygen vacancies in Co_3_O_4_ nanosheets. Excellent electrocatalytical activity of Co_3_O_4_ nanosheet‐decorated zeolite‐templated carbon for ORR can be ascribed to the defects on the Co_3_O_4_ nanosheets and high surface area of carbon supports with uniform pores.

## 5 Conclusions and Outlooks

In summary, recent advances of zeolite‐templated nanocarbon materials were summarized from the aspects of synthesis, structure, morphology, properties, and their applications in energy storage and conversion. Since the first example of zeolite‐templated carbons was reported in 1997, nanocarbon materials templated by zeolites have already extended to CDs, CNTs, graphene, 3D graphene‐like porous carbons, etc. Previously, the applications of zeolite‐templated nanocarbon materials are confined in gas/vapor adsorption. Nowadays, their developments have flourished in the regimes of energy storage and conversion including LIBs, aluminum batteries, MIBs, DIBs, Li–S batteries, Zn–O_2_ batteries, all‐organic proton battery, and fuel cell.

As far as synthesis is concerned, diversity of zeolites brings up the possibilities of preparation of multitudinous nanocarbon materials templated by zeolites. Selection of carbon source is another crucial parameter in structural and morphological modulation and heteroatom doping. Impregnation methods of carbon source also have significant effect on tuning structure and morphology of the nanocarbons. After post‐modification, robust structures can be obtained with a variety of functionalities. Meanwhile, crystalline state gradually disappeared with pores retained. Also, zeolite‐templated nanocarbons are suitable platform to load ultrafine nanostructures such as Co_3_O_4_ nanosheets and Pt nanoparticles, which are of great potential to serve as catalytic active sites.

High specific surface area is not the unique feature of zeolite‐templated nanocarbons, neither high conductivity is. Actually, the crucial characteristics of zeolite‐templated nanocarbons is regularity of pores with plentiful edge sites. More importantly, zeolite‐templated nanocarbons can implement high specific surface area, high conductivity and regularity of pores with plentiful edge sites simultaneously, although there have already been a few reports related to conductive MOFs and COFs, which achieved similar targets.

The energy crisis and environment contamination is threatening the human destiny. Development of environmentally benign energy storage devices is becoming urgent topic for scientists. Zeolite‐templated nanocarbons is playing meaningful parts in energy storage materials: in hydrogen/methane storage, high specific surface area is beneficial for gas/vapor adsorption regardless of the pore structures; besides physisorption, new mechanisms such as hydrogen spillover, hydride‐loading, etc., have been realized by development of, to achieve the target value (>6 wt%) for practical use; as electrode materials, conductive scaffold is the prerequisite, which zeolite‐templated nanocarbons can readily achieve; heteroatom doping increases the electrical conductivity and electrochemical activity of nanocarbons, which could reduce electrolyte resistances and transfer resistances; moreover, exploration of hierarchically ordered porosity can enhance mass transfer without pore blockage, which has been employed as host of active materials in electrode; plus ultrafine nanostructures, enhanced electrocatalytical performance such as ORR can be implemented by synergic effect of nanocarbons and metal oxide nanostructures.

We admit that there have still been a few drawbacks for zeolite‐templated nanocarbons, such as relatively low yield and waste of templates. Contaminations also exist in the process of etching templates due to heavy use of acids. Thus, it is essential to explore the applications of nanocarbons when retaining zeolite templates. Theoretically, the Li capacity of Li‐doped carbon nanotube–zeolite complexes can reach 386 mAh g^−1^ with only a slight increase in the cell volume.^[^ ^280^ ^]^ However, the experimental results have not been reported to date. Fortunately, there have been a few papers related to luminescence properties of CDs confined in zeolites.^[^ ^16^, ^141^, ^160^, ^172^, ^173^, ^174^, ^175^, ^176^, ^177^, ^178^, ^179^ ^]^ Despite multitudinous zeolite‐templated nanocarbon materials have already been reported including CDs, CNTs, graphene nanosheets and 3D graphene‐like porous carbons, diversity of their structure, and morphology still has development space, such as burgeoning carbon nanocages. Furthermore, it can be predicted that zeolite template can be extended to prepare other nanostructures such as black phosphorous,^[^ ^281^ ^]^ perovskite,^[^ ^282^ ^]^ MoS_2_,^[^ ^283^ ^]^ BN,^[^ ^284^, ^285^ ^]^ etc. in future. Finally, we hope the topic of zeolite‐templated nanocarbons continuously develop in other emerging regimes and attract more scientists to join.

## Conflict of Interest

The authors declare no conflict of interest.
